# Definitive Chemoradiotherapy versus Trimodality Therapy for Locally Advanced Esophageal Adenocarcinoma: A Multi-Institutional Retrospective Cohort Study

**DOI:** 10.3390/cancers16162850

**Published:** 2024-08-15

**Authors:** Yang Xu, Ronald Chow, Kyle Murdy, Md Mahsin, Theeva Chandereng, Rishi Sinha, Richard Lee-Ying, Tasnima Abedin, Winson Y. Cheung, Nguyen X. Thanh, Sangjune Laurence Lee

**Affiliations:** 1 Department of Oncology, Tom Baker Cancer Centre, Calgary, AB T2N 4N2, Canada; yang.xu@albertahealthservices.ca (Y.X.);; 2 Department of Oncology, University of Calgary, Calgary, AB T2N 1N4, Canada; 3 Temerty Faculty of Medicine, University of Toronto, Toronto, ON M5S 1A8, Canada; 4 Faculty of Law, University of Calgary, Calgary, AB T2N 1N4, Canada; 5 Precision Oncology Hub, Arnie Charbonneau Cancer Institute, Calgary, AB T2N 4Z6, Canada; md.mahsin@albertahealthservices.ca; 6 Northwell Health, New Hyde Park, NY 11030, USA; 7 Clinical Research Unit, Tom Baker Cancer Centre, Calgary, AB T2N 4N2, Canada; 8 Strategic Clinical Networks, Alberta Health Services, Calgary, AB T5J 3E4, Canada; 9 School of Public Health, University of Alberta, Edmonton, AB T6G 2R3, Canada

**Keywords:** esophageal cancer, adenocarcinoma, definitive chemoradiotherapy, trimodality therapy, neoadjuvant chemoradiotherapy, esophagectomy, metastasis, recurrence, survival

## Abstract

**Simple Summary:**

The combination of chemoradiotherapy followed by surgery (trimodality therapy) is considered standard of care for patients with locally advanced esophageal adenocarcinoma, but many potential candidates for surgery receive curative-intent chemoradiotherapy alone. This study compared the outcomes of patients who received trimodality therapy to those who received curative-intent chemoradiotherapy. Our primary analysis found that trimodality therapy reduced the risk of local tumor recurrences, but we did not detect statistically significant differences in the risks of distant metastases or mortality. These results can help patients and clinicians make informed treatment decisions, although further studies are needed to refine our understanding of the trade-offs between the two treatment strategies.

**Abstract:**

The optimal management of patients with locally advanced esophageal adenocarcinoma is unclear. Neoadjuvant chemoradiotherapy followed by esophagectomy (trimodality therapy) is supported as a standard of care, but definitive chemoradiotherapy is frequently given in practice to patients who may have been surgical candidates. This multi-institutional retrospective cohort study compared the outcomes of consecutive patients diagnosed with stage II to IVA esophageal adenocarcinoma between 2004 and 2018 who planned to undergo trimodality therapy or definitive chemoradiotherapy. A total of 493 patients were included, of whom 435 intended to undergo trimodality therapy and 56 intended to undergo definitive chemoradiotherapy. After a median follow-up of 7.3 years, trimodality therapy was associated with a lower risk of locoregional failure (5-year risk, 30.5% vs. 61.3%; HR, 0.39; 95% CI, 0.24–0.62; p<0.001) but not distant metastases (5-year risk, 58.2% vs. 53.9%; HR, 1.21; 95% CI, 0.77–1.91; p=0.40). There were no differences in overall survival (HR, 0.78; 95% CI, 0.56–1.09; p=0.14) or cancer-specific survival (HR, 0.83; 95% CI, 0.57–1.21; p=0.33). Findings were consistent on propensity score-matched sensitivity analyses. In conclusion, trimodality therapy was associated with a lower risk of locoregional failure, but this did not translate into a significantly lower risk of distant failure or improved survival. Further studies are required to accurately estimate the trade-offs between the two treatment strategies.

## 1. Introduction

Esophageal cancer is the seventh most diagnosed malignancy worldwide, with over 600,000 cases annually [1]. Adenocarcinoma has overtaken squamous cell carcinoma to account for two-thirds of esophageal cancers in high-income countries, and its incidence continues to rise owing to the growing prevalence of its major risk factors, including obesity and gastroesophageal reflux disease [1].

The combination of neoadjuvant chemoradiation and esophagectomy, collectively known as trimodality therapy, is supported as a standard of care for eligible patients with locally advanced esophageal adenocarcinoma [2,3,4,5]. However, esophagectomy remains technically challenging, with a 90-day mortality rate of 4.5% and complication rate of 59% at high-volume centers despite improvements in surgical technique and perioperative care [6]. Moreover, common complications, including anastomotic leaks, conduit failure, pneumonia, and recurrent laryngeal nerve paralysis, may reduce quality of life, predict late mortality, and increase healthcare costs [6,7,8,9]. This has generated interest in the possibility of omitting esophagectomy from the treatment paradigm, with definitive chemoradiotherapy regularly given in practice to patients who may have been candidates for trimodality therapy [10,11].

Two randomized controlled trials have found overall survival after definitive chemoradiotherapy to be equivalent to that of trimodality therapy, but the interpretation of their results is limited by concerns surrounding methodology and a lack of patients with adenocarcinoma [12,13,14]. A recent meta-analysis found that trimodality therapy was associated with higher survival [15]. However, the observational studies that led to this conclusion were susceptible to multiple biases.

This study aims to estimate the relative effectiveness of trimodality therapy compared to definitive chemoradiotherapy for patients with locally advanced esophageal adenocarcinoma.

## 2. Materials and Methods

### 2.1. Patient Population

Following institutional review board approval, we identified patients diagnosed with esophageal adenocarcinoma between 1 January 2004 and 31 December 2018, in Alberta, Canada, using a prospectively maintained provincial database and institutional electronic medical records. Patients were included if there was a plan to undergo trimodality therapy or definitive chemoradiotherapy after multidisciplinary consultation. Patients with American Joint Committee on Cancer (AJCC) 8th edition clinical stage I or IVB disease, who underwent endoscopic mucosal resection, or who had a concurrent primary malignancy were excluded. Patients prescribed a total radiation dose of less than 40 Gy in equivalent dose in 2 Gy fractions (EQD2) using an α/β of 4 Gy were also excluded. This was motivated by the seminal Chemoradiotherapy for Esophageal Cancer Followed by Surgery Study (CROSS) in which patients in the neoadjuvant chemoradiotherapy arm received 41.4 Gy in 23 fractions or 40.0 Gy EQD2 [5]. Finally, patients receiving neoadjuvant chemotherapy alone, neoadjuvant radiotherapy alone, or palliative-intent therapy were excluded.

### 2.2. Patient and Treatment Characteristics

Institutional electronic medical records were reviewed to determine baseline characteristics at the time of diagnosis, including age, sex, comorbidities, Eastern Cooperative Oncology Group (ECOG) performance status, clinical stage, tumor length, treatment intent, planned radiotherapy dose-fractionation, concurrent chemotherapy regimen, and treatment completion status. Charlson Comorbidity Indices (CCIs) were computed using age and comorbidities. Patients not staged using the AJCC 8th edition were retrospectively assigned AJCC 8th edition stages based on initial investigations. Stage II–III NOS was assigned when investigations, including endoscopy and PET-CT, were consistent with either stage II or III, but a lack of endoscopic ultrasonography precluded definitive staging.

Treatment intent, defined herein as the plan to give either trimodality therapy or definitive chemoradiotherapy before the initiation of treatment, was determined using records from initial consultations and multidisciplinary tumor board meetings. Induction and consolidation chemotherapy were not offered with either treatment strategy during the study period. Patients intended for trimodality therapy were re-staged with CT or PET-CT after neoadjuvant chemoradiation and before surgery. All esophagectomies were performed at one of two high-volume esophageal surgical centers. At the time of recurrence, most patients were offered palliative systemic therapy as well as supportive care. Local salvage therapy was rarely used; in particular, none of the patients who underwent definitive chemoradiotherapy received salvage esophagectomy upon locoregional recurrence.

### 2.3. Follow-Up and Outcomes

Patients were followed after treatment with clinical assessments, endoscopy, and cross-sectional imaging per local protocol. Locoregional failure was defined as the first occurrence of locoregional progression or recurrence confirmed by biopsy or cross-sectional imaging. Distant metastatic failure was defined as the first evidence of distant metastases. Time from diagnosis to locoregional failure, distant metastatic failure, and death were determined using provincial records of medical diagnoses, inpatient and outpatient services, and physician billing. The data cutoff was 31 March 2023.

### 2.4. Statistical Analysis

Descriptive statistics of the study population were reported as means with standard deviations for continuous variables and frequencies with percentages for categorical variables. Between-cohort comparisons were performed using Mann–Whitney U tests for continuous variables and $\chi^{2}$ tests for categorical variables. Missing tumor length, CCI, and ECOG performance status were imputed 30 times using multiple imputation by chained equations (MICEs) [16]. Variables used for imputation were age, sex, AJCC 8th edition prognostic stage, tumor length, CCI, ECOG performance status, freedom from locoregional failure, freedom from distant recurrence, and overall survival (event indicator and cumulative hazard).

The primary analysis was based on intention to treat as defined above. For each imputed dataset, multivariable Cox models were used to assess the associations of treatment strategy with locoregional failure, distant metastatic failure, overall survival, and cancer-specific survival. Each model was adjusted for age, sex, prognostic stage, tumor length, CCI, and ECOG performance status. The resulting estimates were pooled according to Rubin’s rules [17].

Per-protocol analyses were also completed wherein patients not undergoing esophagectomy were excluded from the trimodality therapy cohort, and patients not receiving radiotherapy to at least 40.0 Gy EQD2 were excluded from both cohorts. Additionally, the survival of patients who did not undergo esophagectomy as intended was compared to that of patients who completed trimodality therapy per protocol; the former were hypothesized to have poor prognoses but were frequently excluded from the trimodality therapy cohort of observational studies [18,19,20,21,22].

Finally, preprocessing with matching was performed to assess the dependence of estimates on the outcome model [23]. For each imputed intention-to-treat dataset, propensity scores were computed using logistic regression with the following variables: age, sex, prognostic stage, tumor length, CCI, and ECOG performance status. Propensity scores were used to perform 3:1 matching without replacement [24]. The distribution of covariates was evaluated after matching, with absolute standardized differences of less than 0.1, averaged across imputations, considered desirable. Pooled probabilities of recurrence and survival were computed by applying Rubin’s rules to their respective complementary log–log transformations [25,26]. Outcomes were evaluated using multivariable Cox models with the same adjustment variables as the unmatched analyses. These results were pooled with Rubin’s rules and compared to estimates obtained without matching.

All statistical analyses were completed using a two-sided α of 0.05 with R version 4.3.0 (R Foundation for Statistical Computing).

## 3. Results

### 3.1. Study Population

A total of 491 patients were eligible for analysis with a median follow-up of 7.3 years (IQR, 4.7–9.5 years) for overall survival. At the outset, 435 planned to undergo trimodality therapy, while 56 planned to undergo definitive chemoradiotherapy (Table 1). The per-protocol analysis comprised 406 patients, of whom 357 received trimodality therapy, and 49 received definitive chemoradiotherapy. Patients intended for definitive chemoradiotherapy had more advanced disease (stage IV, 34% vs. 15%), more comorbidities (mean CCI, 4.71 vs. 4.16), and were older at the time of diagnosis (mean age, 65.0 years vs. 61.5 years). They were also more likely to have proximal or middle third tumors (10.7% vs. 3.9%).

The treatment characteristics are summarized in Table 2. Cisplatin with 5-fluorouracil was the most common chemotherapeutic regimen given with radiotherapy for patients undergoing definitive chemoradiotherapy (52%), followed by FOLFOX (16%) and carboplatin and paclitaxel (16%). In contrast, carboplatin and paclitaxel was the most common regimen for patients undergoing trimodality therapy (85%). Most patients undergoing definitive chemoradiotherapy were planned for dose escalation beyond 41.4 Gy in 23 fractions (95%), which was the most common dose-fractionation scheme for trimodality therapy (83%). Radiotherapy was terminated prematurely with a dose under 40.0 Gy EQD2 in 15 patients (3%). A total of 67 patients (15%) who intended to undergo trimodality therapy did not have an esophagectomy. The most common reasons to forego esophagectomy were metastatic progression on restaging investigations before surgery (39%), patient refusal after neoadjuvant chemoradiation (19%), evidence of distant metastases on laparoscopy or laparotomy (13%), medical decline after neoadjuvant chemoradiation (13%), unresectable disease at the time of surgery (9%), and patient death before esophagectomy (4%). Thirty-day and ninety-day mortality after esophagectomy were 1.1% and 4.1%, respectively.

### 3.2. Locoregional Failure

**Figure 1:**
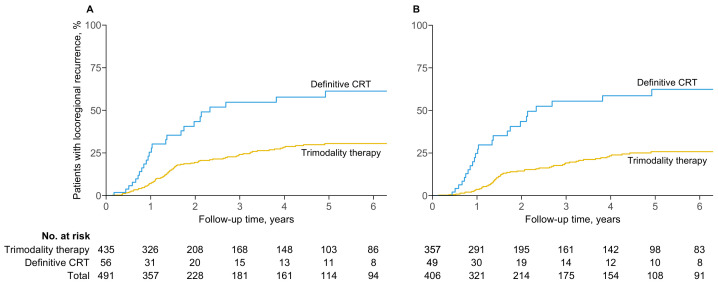
Cumulative probability of locoregional failure by (**A**) intention to treat and (**B**) per protocol.

In the intention-to-treat analysis, the two- and five-year probabilities of locoregional failure for the trimodality therapy cohort were 19.4% (95% CI, 15.0–23.6) and 30.5% (24.7–35.8), respectively, compared to 43.4% (26.2–56.6) and 61.3% (41.8–74.2) for the definitive chemoradiotherapy cohort (Figure 1A). Patients who received trimodality therapy exhibited lower rates of locoregional failure than those who received definitive chemoradiotherapy (adjusted HR, 0.39; 95% CI, 0.24–0.62; p<0.001).

Results were consistent in the per-protocol analysis. The two- and five-year probabilities of locoregional failure were 14.4% (95% CI, 10.2–18.4) and 25.8% (19.8–31.3) for patients who received trimodality therapy, compared to 43.5% (25.8–57.1) and 62.4% (42.2–75.5) for definitive chemoradiotherapy (adjusted HR, 0.28; 95% CI, 0.17–0.47; p<0.001) (Figure 1B).

### 3.3. Distant Metastatic Failure

**Figure 2:**
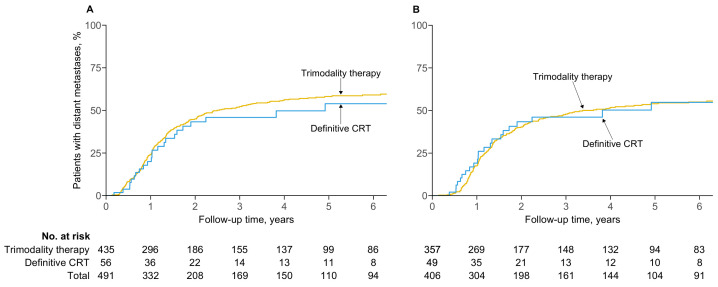
Cumulative probability of distant metastatic failure by (**A**) intention to treat and (**B**) per protocol.

The two- and five-year probabilities of distant metastases in patients intended for trimodality therapy were 45.0% (95% CI, 39.8–49.8) and 58.2% (52.7–63.0), respectively, compared to 43.3% (26.7–56.1) and 53.9% (34.5–67.6) for definitive chemoradiotherapy (Figure 2A). There was no difference in the risk of distant metastases between trimodality therapy and definitive chemoradiotherapy (adjusted HR, 1.21; 95% CI, 0.77–1.91; p=0.40).

In the per-protocol analysis, the two- and five-year probabilities of distant metastases were 40.3% (95% CI, 34.7–45.5) and 54.0% (48.0–59.3) for patients who received trimodality therapy, compared to 43.4% (26.4–56.5) and 54.8% (34.4–68.8) for definitive chemoradiotherapy (Figure 2B). The risk of distant metastases remained similar between the treatment strategies (adjusted HR, 0.98; 95% CI, 0.61–1.58; p=0.94).

### 3.4. Survival

**Figure 3:**
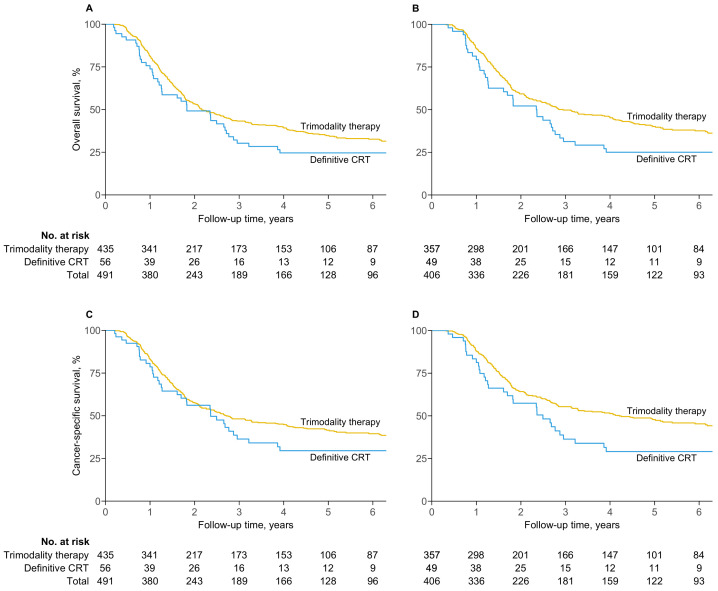
Overall survival and cancer-specific survival. (**A**) Overall survival by intention to treat. (**B**) Overall survival per protocol. (**C**) Cancer-specific survival by intention to treat. (**D**) Cancer-specific survival per protocol.

Median overall survival was 2.2 years (95% CI, 1.9–2.8) among patients intended for trimodality therapy and 1.8 years (1.3–2.8) for definitive chemoradiotherapy (Figure 3A). Median cancer-specific survival was 2.7 years (2.2–4.1) for trimodality therapy and 2.4 years (1.7–3.9) for definitive chemoradiotherapy (Figure 3C). There were no differences in overall survival (adjusted HR, 0.78; 95% CI, 0.56–1.09; p=0.14) or cancer-specific survival (adjusted HR, 0.83; 95% CI, 0.57–1.21; p=0.33) between the two treatment strategies.

In contrast, median overall survival was superior for patients who received trimodality therapy per protocol at 2.9 years (95% CI, 2.4–4.1), compared to 2.4 years (1.6–2.9) for definitive chemoradiotherapy (adjusted HR, 0.69; 95% CI, 0.48–1.00; p=0.047) (Figure 3B). Median per-protocol cancer-specific survival was 4.2 years (3.1–6.9) for trimodality therapy and 2.5 years (1.7–3.9) for definitive chemoradiotherapy (adjusted HR, 0.69; 95% CI, 0.46–1.03; p=0.07) (Figure 3D).

Despite adjustment for baseline characteristics, patients intended for trimodality therapy who did not undergo esophagectomy had significantly worse overall survival (adjusted HR, 3.35; 95% CI, 2.47–4.54; p<0.001) and cancer-specific survival (adjusted HR, 3.71; 95% CI, 2.69–5.13; p<0.001) than those who underwent surgery (Supplemental Figures S1 and S2).

### 3.5. Propensity Score-Matched Analyses

After matching by intended treatment, covariates were well balanced between the trimodality therapy and definitive chemoradiotherapy cohorts with average absolute standardized differences of less than 0.05 for all variables (Supplemental Table S1 and Supplemental Figures S3 and S4). Matched estimates were consistent with unmatched estimates for locoregional failure (adjusted HR, 0.39; 95% CI, 0.22–0.67; p=0.001), distant metastases (adjusted HR, 1.23; 95% CI, 0.74–2.06; p=0.42), overall survival (adjusted HR, 0.78; 95% CI, 0.54–1.13; p=0.19), and cancer-specific survival (adjusted HR, 0.83; 95% CI, 0.55–1.24; p=0.36) (Supplemental Figures S5–S8).

## 4. Discussion

We found that trimodality therapy was associated with a lower risk of locoregional failure and a similar risk of distant metastatic failure compared to definitive chemoradiotherapy in patients with locally advanced esophageal adenocarcinoma. There were no significant differences in overall or cancer-specific survival in the primary intention-to-treat analysis, but trimodality therapy was associated with superior overall survival in the per-protocol analysis.

Despite the growing incidence of esophageal adenocarcinoma, few trials have compared definitive chemoradiotherapy and trimodality therapy. The Fédération Francophone de Cancérologie Digestive 9102 study is the only completed randomized controlled trial that included patients with esophageal adenocarcinoma [13]. Its results were inconclusive; two-year overall survival was non-inferior among those who received definitive chemoradiotherapy in the intention-to-treat analysis but not the per-protocol analysis using a margin of 10%. Similar to our findings, the two-year risk of locoregional failure was lower among patients who received trimodality therapy, but there was no difference in the risk of distant failure. However, only 29 patients had esophageal adenocarcinoma, casting uncertainty on the generalizability of these findings. Furthermore, most patients received a split-course radiotherapy regimen with a low biologically effective dose. Finally, the 90-day operative mortality rate was 9.3%, more than double that of a large international series [6].

By contrast, observational studies have consistently reported better outcomes with trimodality therapy [18,19,20,21,22]. In a recent meta-analysis, trimodality therapy was associated with markedly better overall survival in studies that included only patients with adenocarcinoma (HR, 0.54; 95% CI, 0.46–0.65) and studies that combined squamous cell carcinoma and adenocarcinoma (HR, 0.55; 95% CI, 0.43–0.69) [15]. However, these studies had important limitations. Established prognostic factors were frequently unavailable, leading to a high risk of confounding. Studies based on registry data did not capture radiotherapy doses, potentially resulting in the inclusion of patients who received suboptimal treatment or whose treatment intent was palliative. Additionally, none of the studies performed an intention-to-treat analysis. Patients intended for trimodality therapy frequently forego surgery for reasons including clinical decline or metastatic progression. This affected 15% of patients in the present study and 17% in another large series, and the prognosis of such patients was dismal in both studies [27]. Failure to include these patients resulted in selection bias in favor of trimodality therapy. Compounding the problem, their treatment is susceptible to misclassification as definitive chemoradiotherapy, thereby biasing outcomes against definitive chemoradiotherapy. Finally, these studies were subject to immortal time bias that was either not considered or corrected using methods predicated on strong assumptions about the timing of surgery that do not reflect the variations seen in practice [22].

Accurate survival estimates are imperative to informed treatment decisions, as patients are willing to sacrifice overall survival to avoid esophagectomy [28]. Our results suggest that the survival benefit conferred by trimodality therapy may be smaller than estimated in other observational studies [18,19,21,22]. This difference could be partly explained by biases in previous studies. Indeed, excluding patients who did not complete their intended treatment in our per-protocol analysis improved overall survival for trimodality therapy. While this study upholds trimodality therapy as the standard of care to maximize locoregional control and overall survival, it also supports definitive chemoradiotherapy as a viable alternative for selected patients who are reluctant or unable to undergo surgery.

Our finding of a higher risk of locoregional failure among patients treated with definitive chemoradiotherapy without a corresponding increase in distant metastases suggests that any difference in mortality is likely to be driven by complications of locoregional failure. This can potentially be mitigated with salvage esophagectomy, which was not performed on any of the patients in our definitive chemoradiotherapy cohort. Although salvage esophagectomy was associated with a high risk of morbidity and mortality in early studies [29,30], contemporary approaches have improved outcomes to levels comparable to immediate esophagectomy [31,32,33].

Definitive chemoradiotherapy followed by salvage esophagectomy for isolated local failures may represent an attractive treatment strategy. Similar to locally advanced rectal cancer, where nonoperative management is increasingly adopted, the initially modest rate of pathological complete response in patients with esophageal adenocarcinoma may increase to over 40% with longer time intervals from neoadjuvant chemoradiation to surgery [34,35]. We found that approximately one-third of long-term definitive chemoradiotherapy survivors do not experience locoregional failure. Furthermore, over half of the patients who underwent trimodality therapy developed distant metastases, rendering their esophagectomies futile. The omission of planned surgery also furnishes opportunities to condense and escalate systemic therapy, an area of investigation whose importance is underscored by the high rate of distant metastases in this study and the encouraging outcomes of perioperative 5-FU, leucovorin, oxaliplatin, and docetaxel (FLOT) in the ESOPEC trial [36,37]. Crucially, our results suggest that delaying esophagectomy until locoregional failure may not substantially increase the risk of distant metastases, although caution is warranted in extrapolating these findings to patients treated with de-escalated neoadjuvant chemoradiotherapy regimens [38]. The comparison of active surveillance to planned esophagectomy after complete clinical response to neoadjuvant chemoradiotherapy is the subject of the ongoing Surgery As Needed for Oesophageal cancer (SANO) trial, which has enrolled patients with both adenocarcinoma and squamous cell carcinoma [39]. An initial finding of non-inferior two-year overall survival with active surveillance using a 15% margin was recently reported [33], but longer follow-up is required.

This study has several strengths. We performed both intention-to-treat and per-protocol analyses without the risk of treatment misclassification. The intention-to-treat analysis further eliminates immortal time bias arising from the longer event-free time required of patients undergoing surgery. There was no evidence of suboptimal outcomes in either treatment cohort. Notably, our 30-day and 90-day operative mortality rates of 1.1% and 4.1%, respectively, compare favorably to that of high-volume esophageal surgical centers at 2.4% and 4.5% [6]. The survival of the trimodality therapy cohort in this study is shorter than that of the CROSS trial, but this likely reflects the stringent eligibility criteria of the trial and consequent differences in patient age, comorbidities, performance status, and cancer stage [5,40]. Outcomes of patients who underwent trimodality therapy in this study are reassuringly consistent with those reported by large registries [19,21,22]. Finally, multiple endpoints, adjusted for important prognostic variables, were examined to characterize the patterns of failure between the two cohorts. These results were compared to estimates obtained from propensity score-matched analyses that reduce the risk of bias if the outcome model is misspecified [23,41]. The consistency of estimates from the two statistical methods strengthens our conclusions.

There are important limitations to this study. The determination of surgical candidacy involves nuance that is unlikely to be fully captured by the variables in this study. As such, the propensity and outcome models cannot be expected to eliminate selection bias entirely. Additionally, the unequal cohort sizes of this study reduce its ability to detect small differences in outcomes. We did not compare the quality of life experienced by patients in the two cohorts. Early results from the SANO trial suggest that the the omission of planned esophagectomy can improve short-term quality of life [33]; further studies on this subject would help to inform patient expectations. Lastly, none of the patients in this study received immunotherapy. Nivolumab is now routinely given at our institutions based on the CheckMate 577 trial [42], but outcomes for these patients have yet to mature. The changing landscape of systemic therapy for locally advanced esophageal cancer will likely continue to pose challenges to prospective and retrospective studies alike.

## 5. Conclusions

In patients with locally advanced esophageal adenocarcinoma, trimodality therapy was associated with a lower risk of locoregional failure than definitive chemoradiotherapy. However, this did not translate into a statistically significant reduction in the risk of distant metastases or improvement in survival in the primary intention-to-treat analysis. A survival advantage might exist for trimodality therapy based on the per-protocol analysis, but this finding is subject to a risk of bias favoring trimodality therapy, and any such advantage may be smaller than estimated in previous observational studies. Further studies are required to provide patients with accurate estimates of the trade-offs of foregoing esophagectomy.

## Figures and Tables

**Table 1:** Baseline patient characteristics.

	Trimodality Therapy	Definitive Chemoradiotherapy	
**Characteristic**	**(** ***n*** ** = 435)**	**(** ***n*** ** = 56)**	p ** Value**
Age at diagnosis, years			
Mean (SD)	61.5 (9.4)	65.0 (9.5)	0.02 ^1^
<50	43 (9.9%)	3 (5.4%)	
50 to 59	139 (32.0%)	13 (23.2%)	
60 to 69	161 (37.0%)	21 (37.5%)	
70 to 79	83 (19.1%)	16 (28.6%)	
≥80	9 (2.1%)	3 (5.4%)	
Sex			
Male	381 (87.6%)	47 (83.9%)	0.58 ^2^
Female	54 (12.4%)	9 (16.1%)	
AJCC 8th edition prognostic stage			
II	25 (5.7%)	7 (12.5%)	<0.001 ^2^
III	253 (58.2%)	24 (42.9%)	
II or III (no EUS)	92 (21.1%)	6 (10.7%)	
IVA	65 (14.9%)	19 (33.9%)	
Tumor location			
Proximal third	1 (0.2%)	2 (3.6%)	0.005 ^2^
Middle third	16 (3.7%)	4 (7.1%)	
Distal third or GEJ	418 (96.1%)	50 (89.3%)	
Tumor length, centimeters			
Mean (SD)	5.62 (2.64)	6.04 (3.48)	0.54 ^1^
<4	70 (16.1%)	8 (14.3%)	
[4, 6)	119 (27.4%)	16 (28.6%)	
[6, 8)	62 (14.3%)	10 (17.9%)	
[8, 10)	40 (9.2%)	3 (5.4%)	
≥10	30 (6.9%)	4 (7.1%)	
Unknown	114 (26.2%)	15 (26.8%)	
Tumor grade			
1	17 (3.9%)	2 (3.6%)	0.41 ^2^
2	112 (25.7%)	16 (28.6%)	
3	141 (32.4%)	19 (33.9%)	
Unknown	165 (37.9%)	19 (33.9%)	
Charlson Comorbidity Index			
Mean (SD)	4.16 (1.31)	4.71 (1.42)	0.007 ^1^
2	32 (7.4%)	1 (1.8%)	
3	105 (24.1%)	9 (16.1%)	
4	134 (30.8%)	15 (26.8%)	
5	104 (23.9%)	15 (26.8%)	
≥6	52 (12.0%)	12 (21.4%)	
Unknown	8 (1.8%)	4 (7.1%)	
ECOG performance status			
Mean (SD)	0.65 (0.60)	0.77 (0.70)	0.29 ^1^
0	178 (40.9%)	20 (35.7%)	
1	220 (50.6%)	24 (42.9%)	
2	29 (6.7%)	8 (14.3%)	
Unknown	8 (1.8%)	4 (7.1%)	

Abbreviations: SD, standard deviation; AJCC, American Joint Committee on Cancer; EUS, endoscopic ultrasound; GEJ, gastroesophageal junction; [x, y), the interval of tumor lengths of at least x and less than y. ^1^ Mann–Whitney U test. ^2^ χ^2^ test.

**Table 2:** Treatment characteristics.

	Trimodality Therapy	Definitive Chemoradiotherapy	
**Characteristic**	**(** ***n*** ** = 435)**	**(** ***n*** ** = 56)**	p ** Value**
Concurrent chemotherapy regimen			
Cisplatin and 5-FU	44 (10.1%)	29 (51.8%)	<0.001 ^1^
Carboplatin and paclitaxel	369 (84.8%)	9 (16.1%)	
FOLFOX	1 (0.2%)	9 (16.1%)	
Other	13 (3.0%)	4 (7.1%)	
Unknown	8 (1.8%)	5 (8.9%)	
Planned radiotherapy dose-fractionation			
Mean EQD2, Gy (SD)	41.29 (3.11)	49.07 (3.00)	<0.001 ^2^
41.4 Gy in 23 fractions	361 (83.0%)	3 (5.4%)	
45 Gy in 25 fractions	24 (5.5%)	2 (3.6%)	
50 Gy in 25 fractions	37 (8.5%)	39 (69.6%)	
50.4 Gy in 28 fractions	9 (2.1%)	8 (14.3%)	
Other	4 (0.9%)	4 (7.1%)	
Dose delivered			
Mean EQD2, Gy (SD)	40.92 (4.19)	47.40 (7.66)	<0.001 ^2^
EQD2 <40 Gy	12 (2.8%)	3 (5.4%)	
EQD2 ≥40 Gy	423 (97.2%)	53 (94.6%)	

Abbreviations: 5-FU, 5-fluorouracil; FOLFOX, 5-fluorouracil and leucovorin and oxaliplatin; SD, standard deviation; EQD2, equivalent dose in 2 Gy fractions with α/β of 4.0 Gy. ^1^ χ^2^ test. ^2^ Mann–Whitney U test.

## Data Availability

The data analyzed in the present study are not publicly available because of privacy restrictions and confidentiality concerns. They can be obtained from the corresponding author upon reasonable request.

## Supplementary Materials

The following supporting information can be downloaded at: https://www.mdpi.com/article/10.3390/cancers16162850/s1, Table S1: Mean baseline-imputed patient characteristics across all imputations, before and after matching; Figure S1: Overall survival of patients intended for trimodality therapy by surgical status; Figure S2: Cancer-specific survival of patients intended for trimodality therapy by surgical status; Figure S3: Standardized differences for matched variables, before and after propensity score matching; Figure S4: Absolute standardized differences for matched variables, before and after propensity score matching; Figure S5: Propensity score-matched cumulative probability of local failure; Figure S6: Propensity score-matched cumulative probability of distant metastatic failure; Figure S7: Propensity score-matched overall survival; Figure S8: Propensity score-matched cancer-specific survival.
