# Supplementary material for: Definitive Chemoradiotherapy versus Trimodality Therapy for Locally Advanced Esophageal Adenocarcinoma: A Multi-Institutional Retrospective Cohort Study
Source: Cancers (Basel). 2024 Aug 15;16(16):2850. doi: 10.3390/cancers16162850 (PMC11353245; doi:10.3390/cancers16162850)
Supplement: Supplementary file 1 [file cancers-16-02850-s001.zip › cancers-3112913-supplementary.pdf]

Supplementary Materials: Definitive Chemoradiotherapy versus Trimodality Therapy for Locally Advanced Esophageal Adenocarcinoma: A Multi-institutional Retrospective Cohort Study

Table S1. Mean baseline-imputed patient characteristics across all imputations, before and after matching.
Figure S1. Overall survival of patients intended for trimodality therapy by surgical status.
Figure S2. Cancer-specific survival of patients intended for trimodality therapy by surgical status.
Figure S3. Standardized differences for matched variables, before and after propensity score matching.
Figure S4. Absolute standardized differences for matched variables, before and after propensity score matching.
Figure S5. Propensity score-matched cumulative probability of local failure.
Figure S6. Propensity score-matched cumulative probability of distant metastatic failure.
Figure S7. Propensity score-matched overall survival.
Figure S8. Propensity score-matched cancer-specific survival.

1. Supplemental Tables

Table S1. Mean baseline-imputed patient characteristics across all imputations, before and after matching.

	Imputed, without Matching			Imputed, after Matching		
**Characteristic**	**TMT (n = 435)**	**Definitive CRT (n = 56)**	**Mean SMD (Mean ASMD)**	**TMT (n = 168)**	**Definitive CRT (n = 56)**	**Mean SMD (Mean ASMD)**
Age at diagnosis, years						
Mean (SD)	61.5 (9.4)	65.0 (9.5)	0.362 (0.362)	64.7 (9.0)	65.0 (9.5)	0.028 (0.038)
Sex						
Male	381 (87.6%)	47 (83.9%)	−0.100 (0.100)	142.9 (85.1%)	47 (83.9%)	−0.031 (0.044)
Female	54 (12.4%)	9 (16.1%)	0.100 (0.100)	25.1 (14.9%)	9 (16.1%)	0.031 (0.044)
AJCC 8th edition stage						
II	25 (5.7%)	7 (12.5%)	0.204 (0.204)	18.8 (11.2%)	7 (12.5%)	0.040 (0.041)
III	253 (58.2%)	24 (42.9%)	−0.309 (−0.309)	73.1 (43.5%)	24 (42.9%)	−0.013 (0.035)
II or III (no EUS)	92 (21.1%)	6 (10.7%)	−0.337 (−0.337)	18.8 (11.2%)	6 (10.7%)	−0.016 (0.043)
IVA	65 (14.9%)	19 (33.9%)	0.401 (0.401)	57.3 (34.1%)	19 (33.9%)	−0.003 (0.021)
Tumor length, centimeters						
Mean (SD)	5.60 (2.65)	5.83 (3.28)	0.071 (0.077)	5.78 (2.83)	5.83 (3.28)	0.017 (0.035)
Charlson Comorbidity Index						
Mean (SD)	4.16 (1.32)	4.70 (1.43)	0.373 (0.373)	4.68 (1.43)	4.70 (1.43)	0.016 (0.027)
ECOG performance status						
Mean (SD)	0.65 (0.60)	0.76 (0.70)	0.160 (0.160)	0.75 (0.60)	0.76 (0.70)	0.027 (0.036)

Abbreviations: TMT, trimodality therapy; CRT, chemoradiotherapy; SMD, standardized (mean) difference; ASMD, absolute standardized (mean) difference.

2. Supplemental Figures

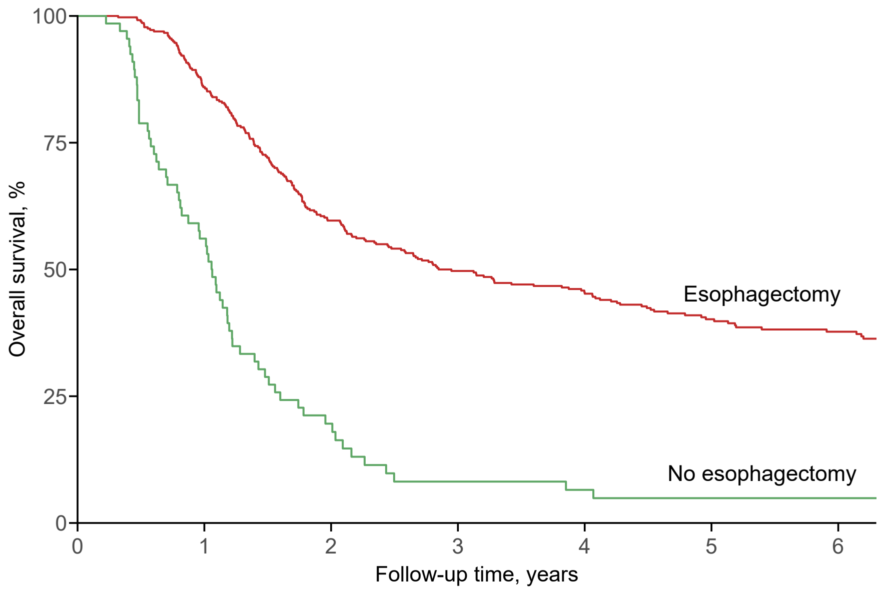

No. at risk							
Esophagectomy	368	304	205	168	149	103	85
No esophagectomy	67	37	12	5	4	3	2
Total	435	341	217	173	153	106	87

Figure S1. Overall survival of patients intended for trimodality therapy by surgical status.

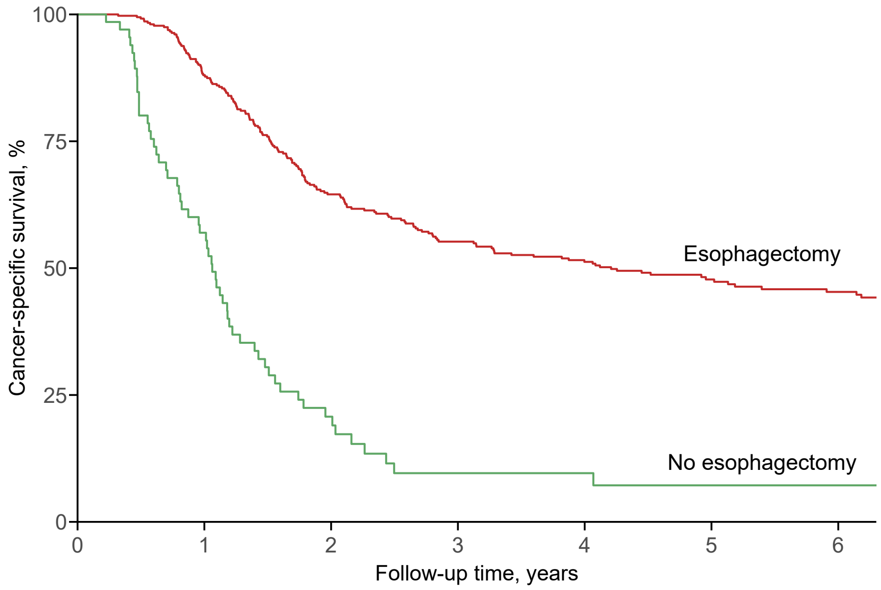

No. at risk							
Esophagectomy	368	304	205	168	149	103	85
No esophagectomy	67	37	12	5	4	3	2
Total	435	341	217	173	153	106	87

Figure S2. Cancer-specific survival of patients intended for trimodality therapy by surgical status.

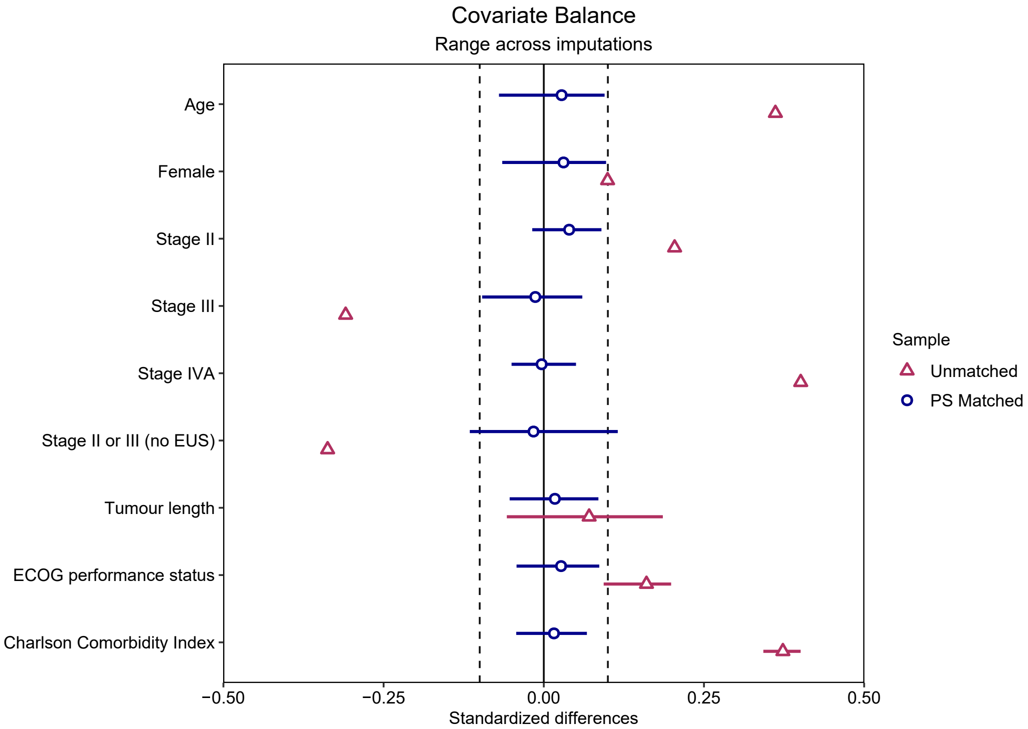

Figure S3. Standardized (mean) differences (SMDs) for matched variables, before and after propensity score matching. Triangles and circles denote the mean SMD of the 30 imputed datasets before and after propensity score matching, respectively. Error bars denote the range of SMDs across all 30 imputed datasets.

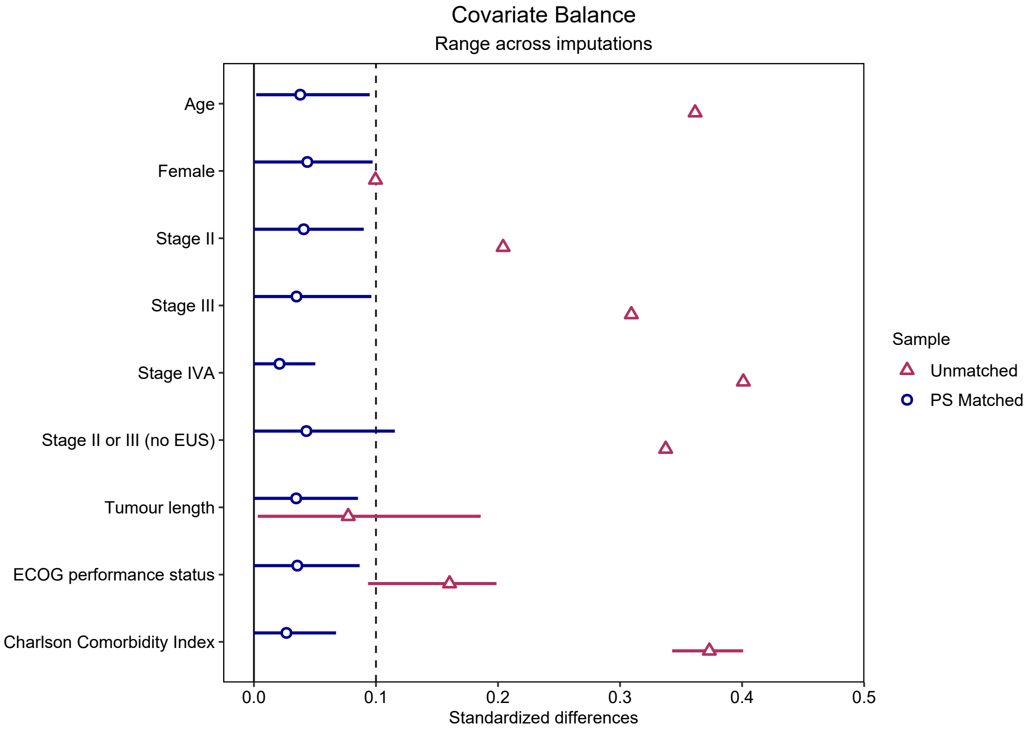

Figure S4. Absolute standardized (mean) differences (SMDs) for matched variables, before and after propensity score matching. Triangles and circles denote the mean absolute SMD of the 30 imputed datasets before and after propensity score matching, respectively. Error bars denote the range of absolute SMDs across all 30 imputed datasets.

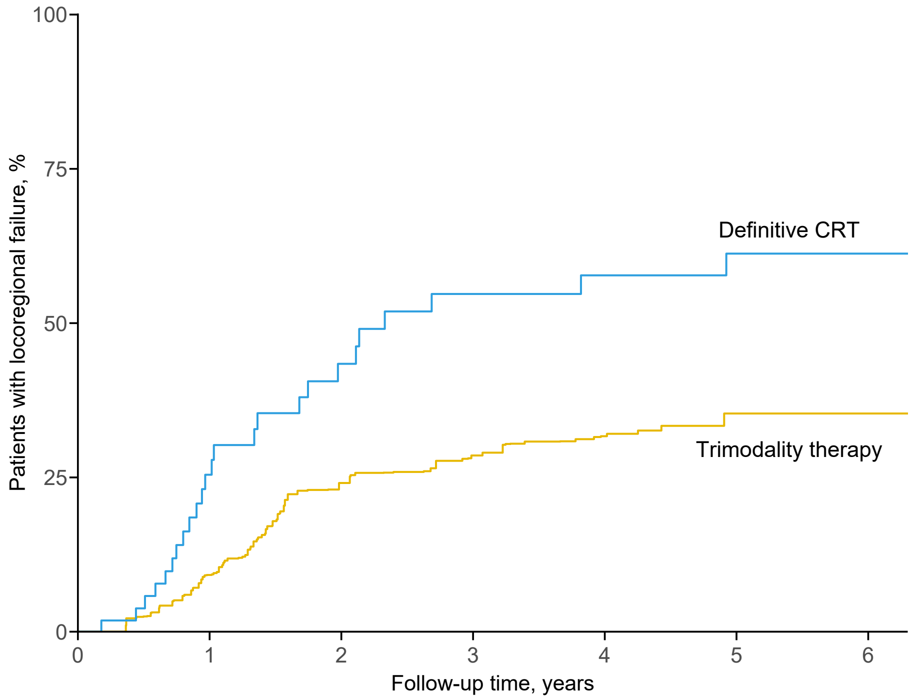

Figure S5. Propensity score-matched cumulative probability of local failure.

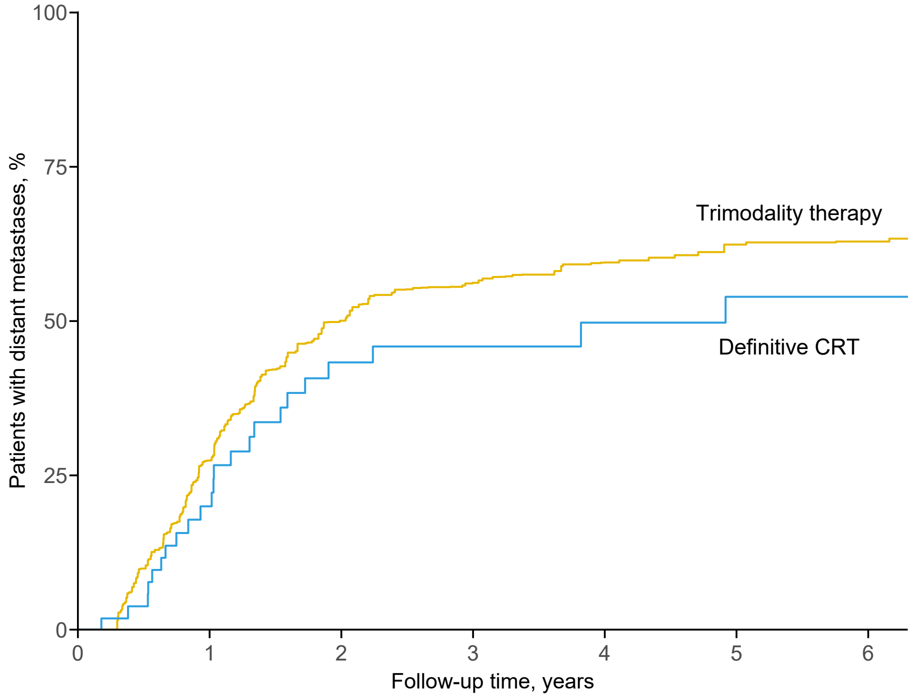

Figure S6. Propensity score-matched cumulative probability of distant metastatic failure.

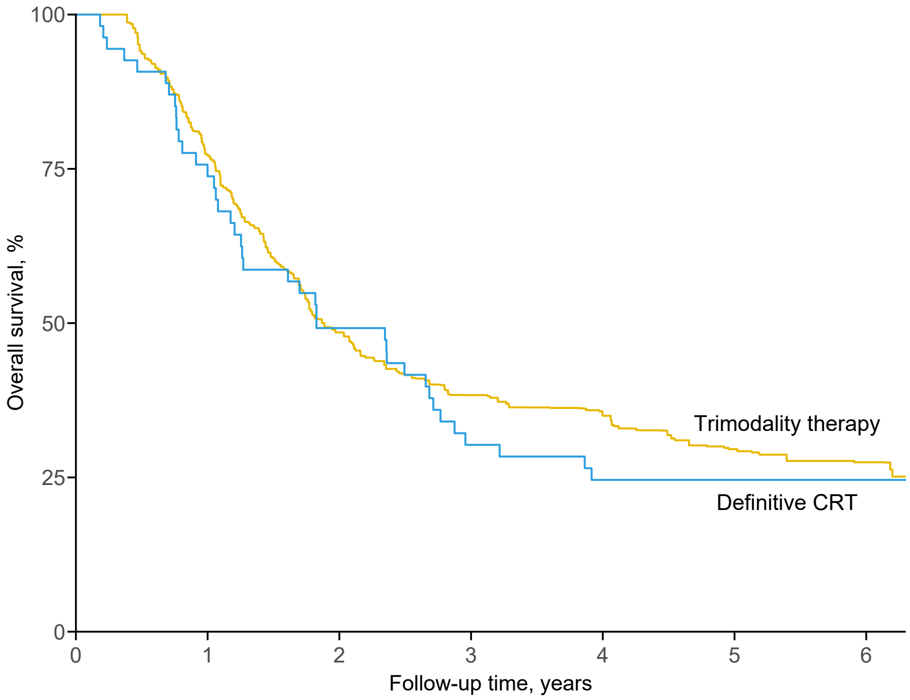

Figure S7. Propensity score-matched overall survival.

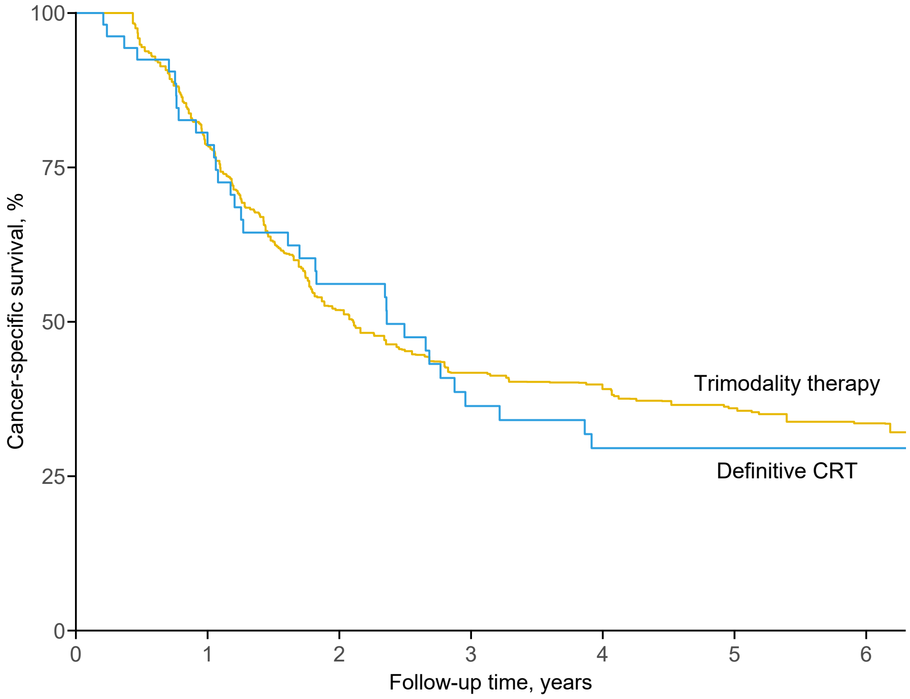

Figure S8. Propensity score-matched cancer-specific survival.
