# Supplementary figures and images for: Definitive Chemoradiotherapy versus Trimodality Therapy for Locally Advanced Esophageal Adenocarcinoma: A Multi-Institutional Retrospective Cohort Study
Source: Cancers (Basel). 2024 Aug 15;16(16):2850. doi: 10.3390/cancers16162850 (PMC11353245; doi:10.3390/cancers16162850)

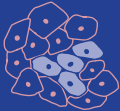

*cancers*

Supplement: Supplementary file 1 [file cancers-16-02850-s001.zip › Definitions/cancers-logo-eps-converted-to.pdf]

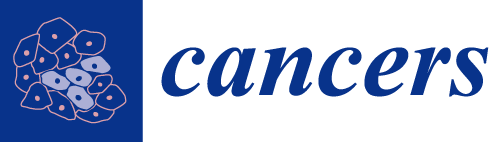

Supplement: Supplementary file 1 [file cancers-16-02850-s001.zip › Definitions/cancers-logo.png]

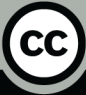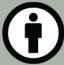

BY

Supplement: Supplementary file 1 [file cancers-16-02850-s001.zip › Definitions/logo-ccby-eps-converted-to.pdf]

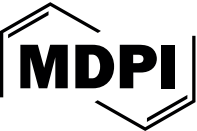

Supplement: Supplementary file 1 [file cancers-16-02850-s001.zip › Definitions/logo-mdpi-eps-converted-to.pdf]

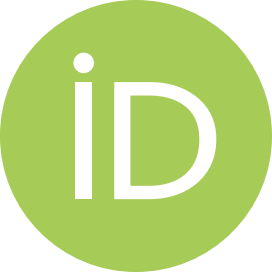

Supplement: Supplementary file 1 [file cancers-16-02850-s001.zip › Definitions/logo-orcid.pdf]

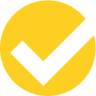

check for  
updates

Supplement: Supplementary file 1 [file cancers-16-02850-s001.zip › Definitions/logo-updates-eps-converted-to.pdf]

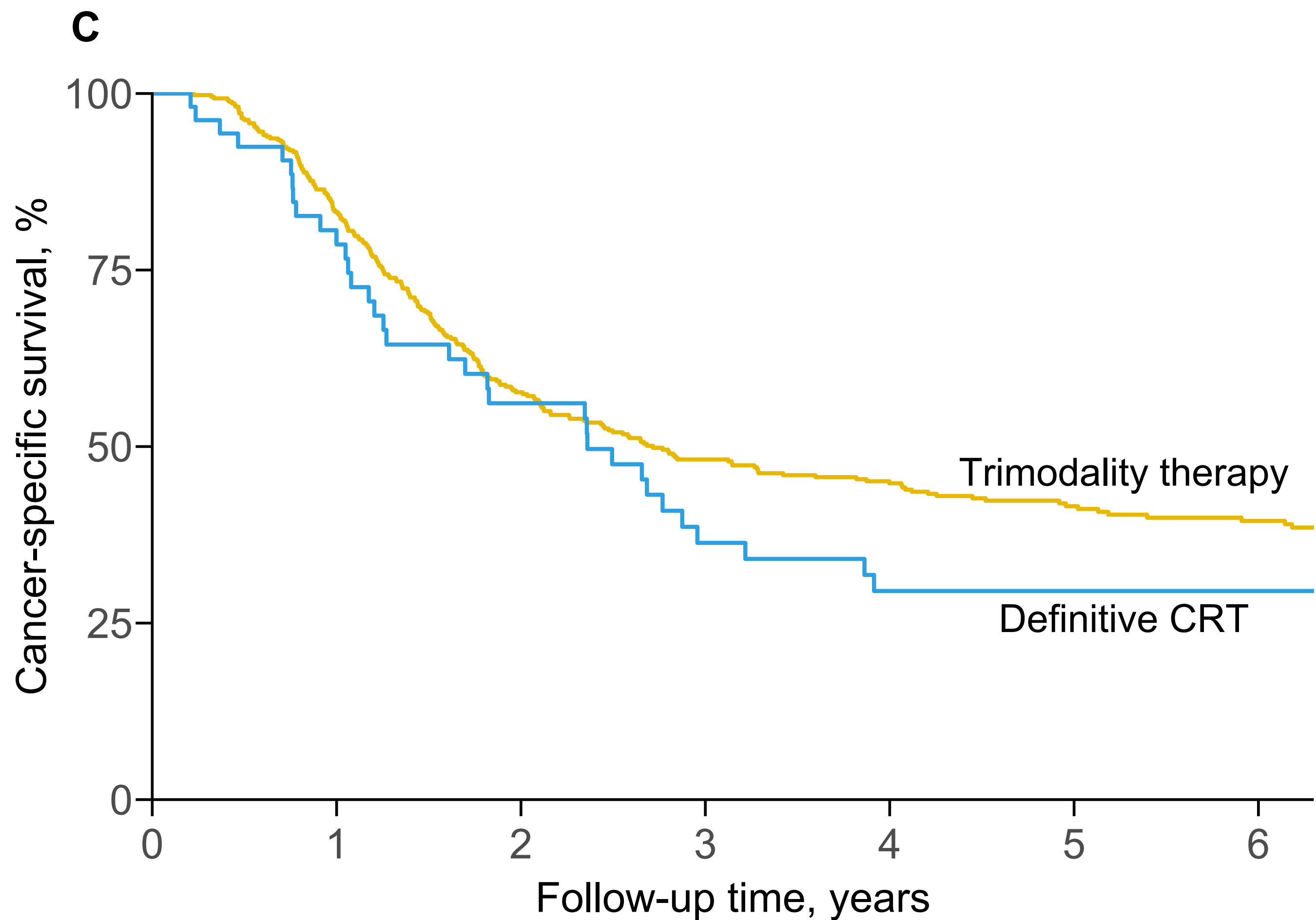

**No. at risk**

|                     |     |     |     |     |     |     |    |
|---------------------|-----|-----|-----|-----|-----|-----|----|
| Trimodality therapy | 435 | 341 | 217 | 173 | 153 | 106 | 87 |
| Definitive CRT      | 56  | 39  | 26  | 16  | 13  | 12  | 9  |
| Total               | 491 | 380 | 243 | 189 | 166 | 128 | 96 |

Supplement: Supplementary file 1 [file cancers-16-02850-s001.zip › Figures/CSS-eps-converted-to.pdf]

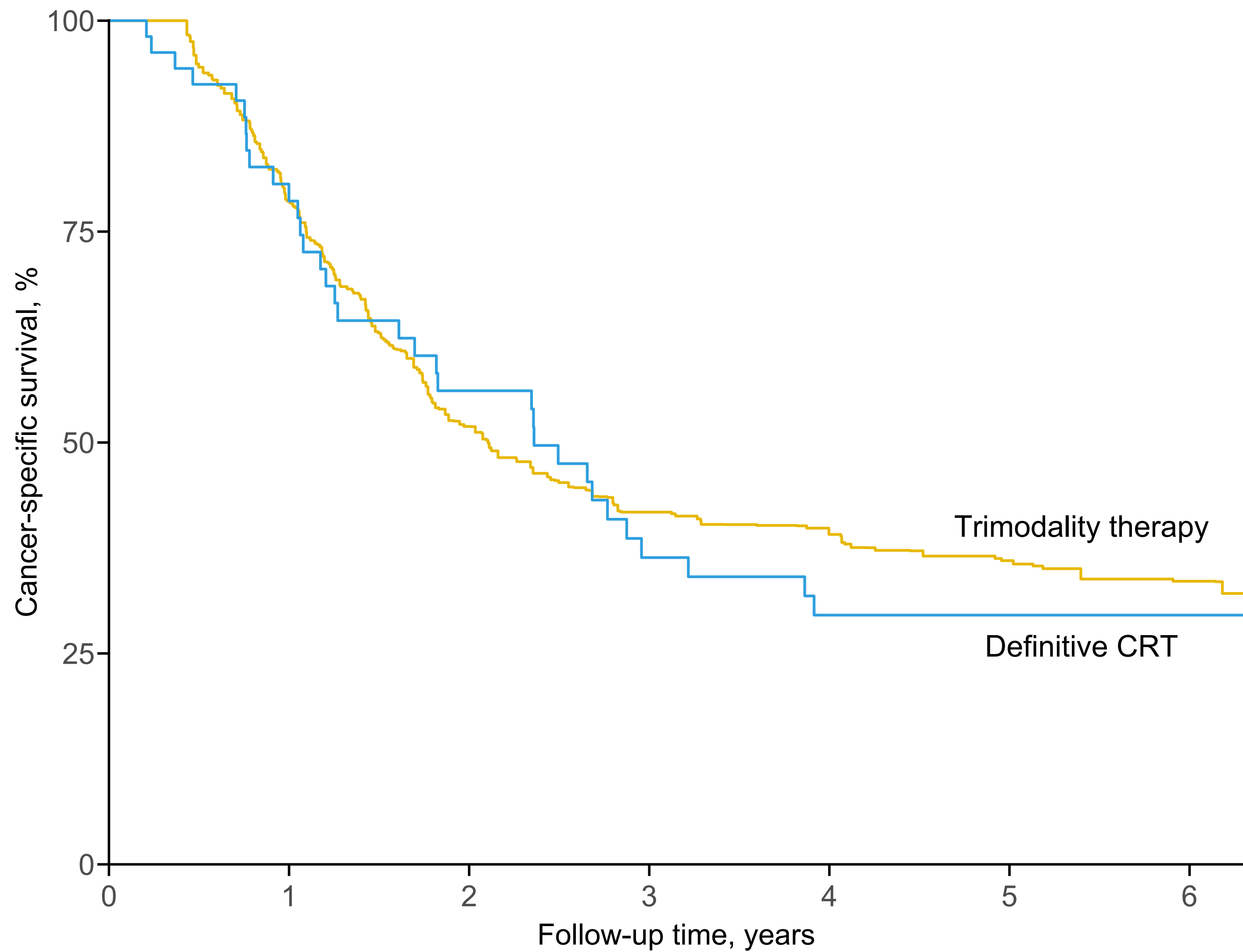

Supplement: Supplementary file 1 [file cancers-16-02850-s001.zip › Figures/CSS_pooled-eps-converted-to.pdf]

**D**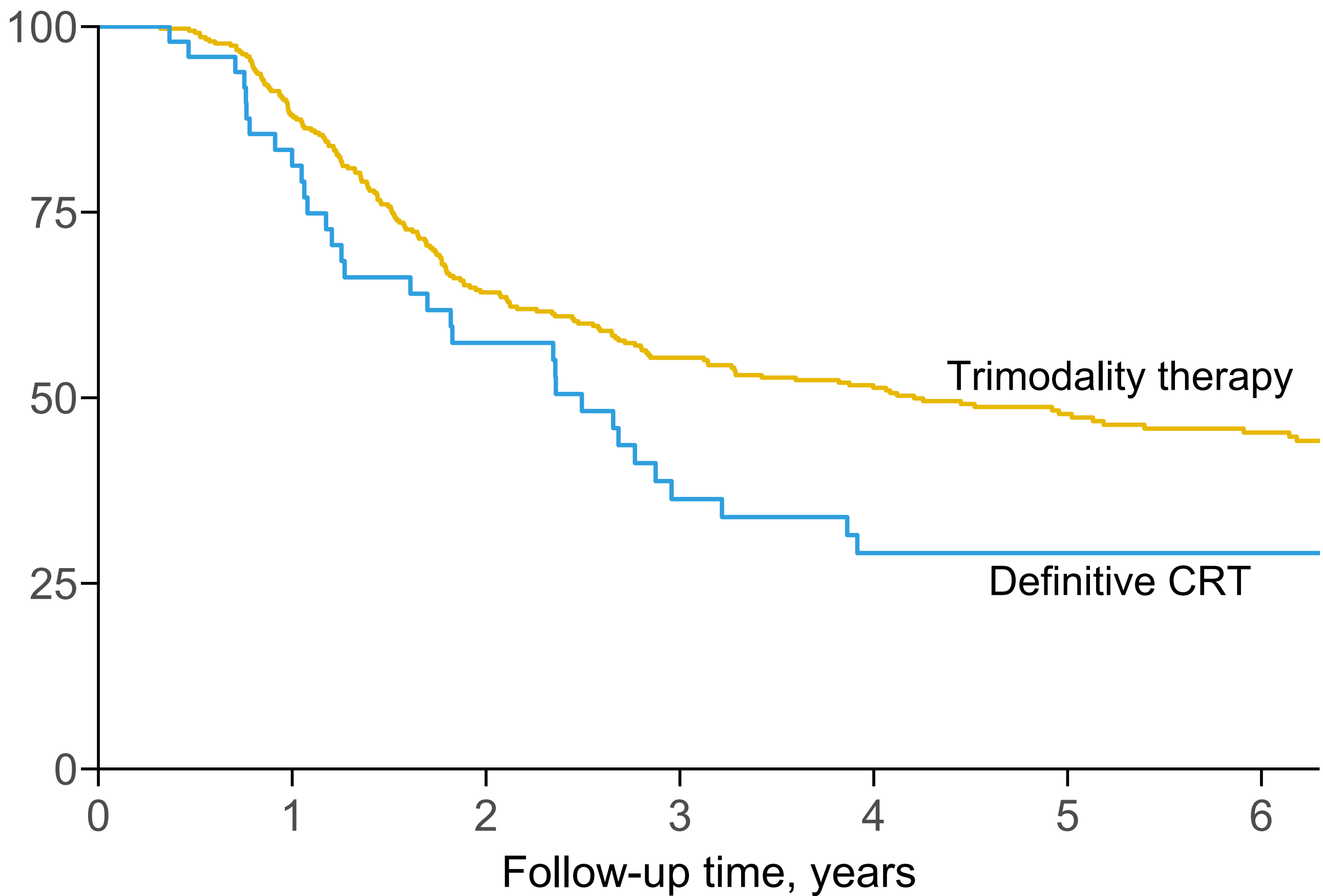

357

298

201

166

147

101

84

49

38

25

15

12

11

9

406

336

226

181

159

122

93

Supplement: Supplementary file 1 [file cancers-16-02850-s001.zip › Figures/CSS_PP_abb-eps-converted-to.pdf]

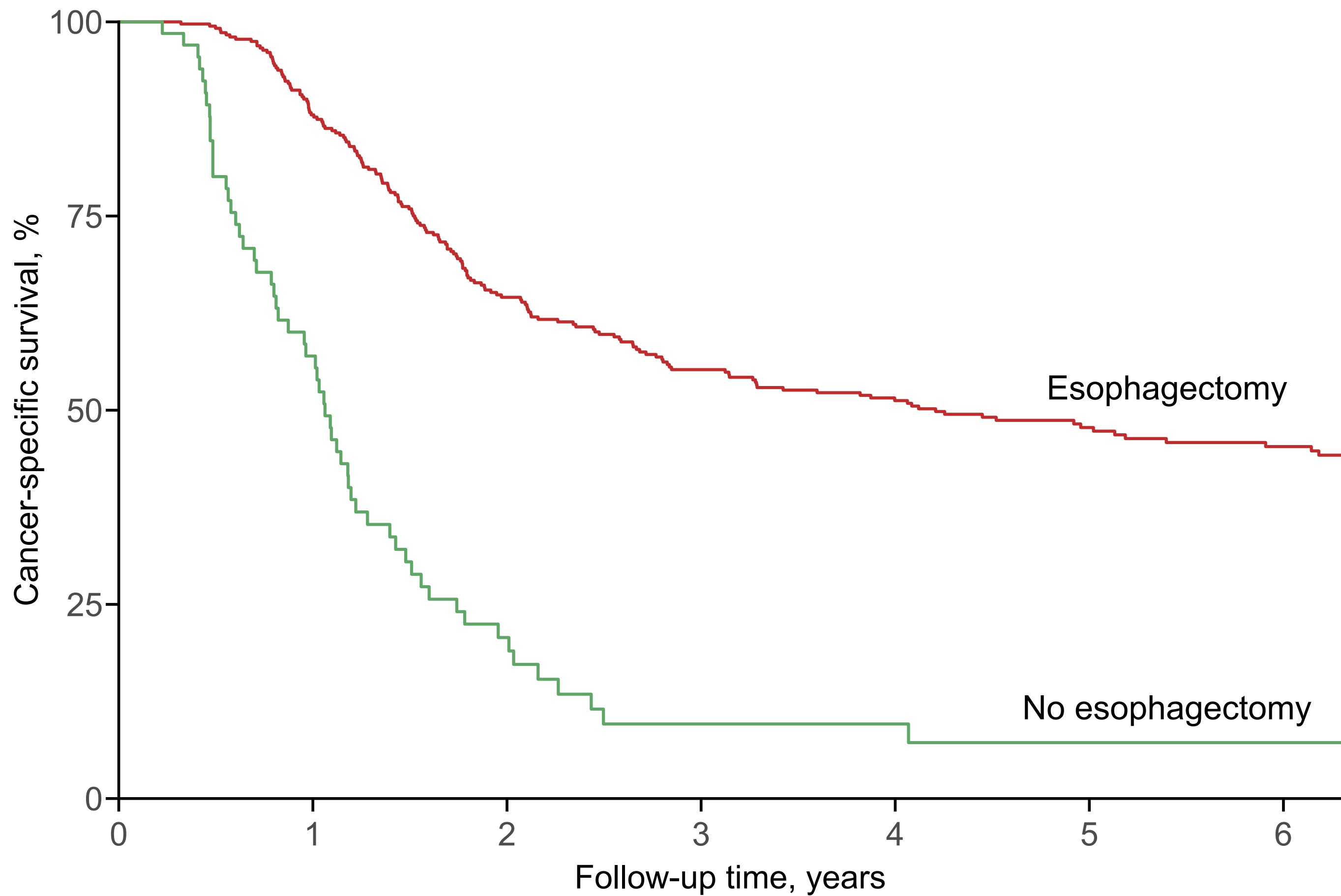

| No. at risk      |     |     |     |     |     |     |    |
|------------------|-----|-----|-----|-----|-----|-----|----|
| Esophagectomy    | 368 | 304 | 205 | 168 | 149 | 103 | 85 |
| No esophagectomy | 67  | 37  | 12  | 5   | 4   | 3   | 2  |
| Total            | 435 | 341 | 217 | 173 | 153 | 106 | 87 |

Supplement: Supplementary file 1 [file cancers-16-02850-s001.zip › Figures/CSS_trimodal_intent_surgery-eps-converted-to.pdf]

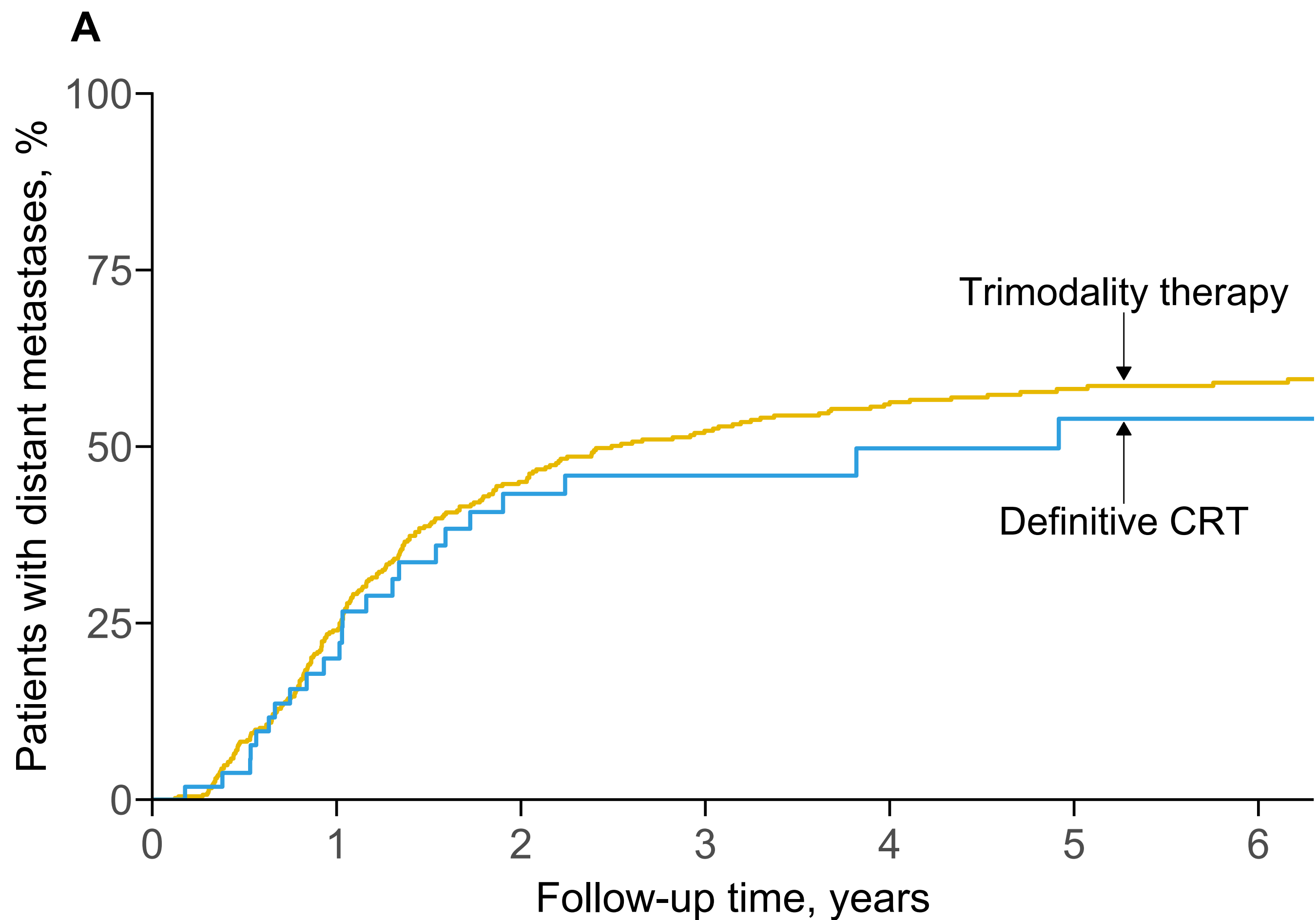

**No. at risk**

|                     |     |     |     |     |     |     |    |
|---------------------|-----|-----|-----|-----|-----|-----|----|
| Trimodality therapy | 435 | 296 | 186 | 155 | 137 | 99  | 86 |
| Definitive CRT      | 56  | 36  | 22  | 14  | 13  | 11  | 8  |
| Total               | 491 | 332 | 208 | 169 | 150 | 110 | 94 |

Supplement: Supplementary file 1 [file cancers-16-02850-s001.zip › Figures/DMF-eps-converted-to.pdf]

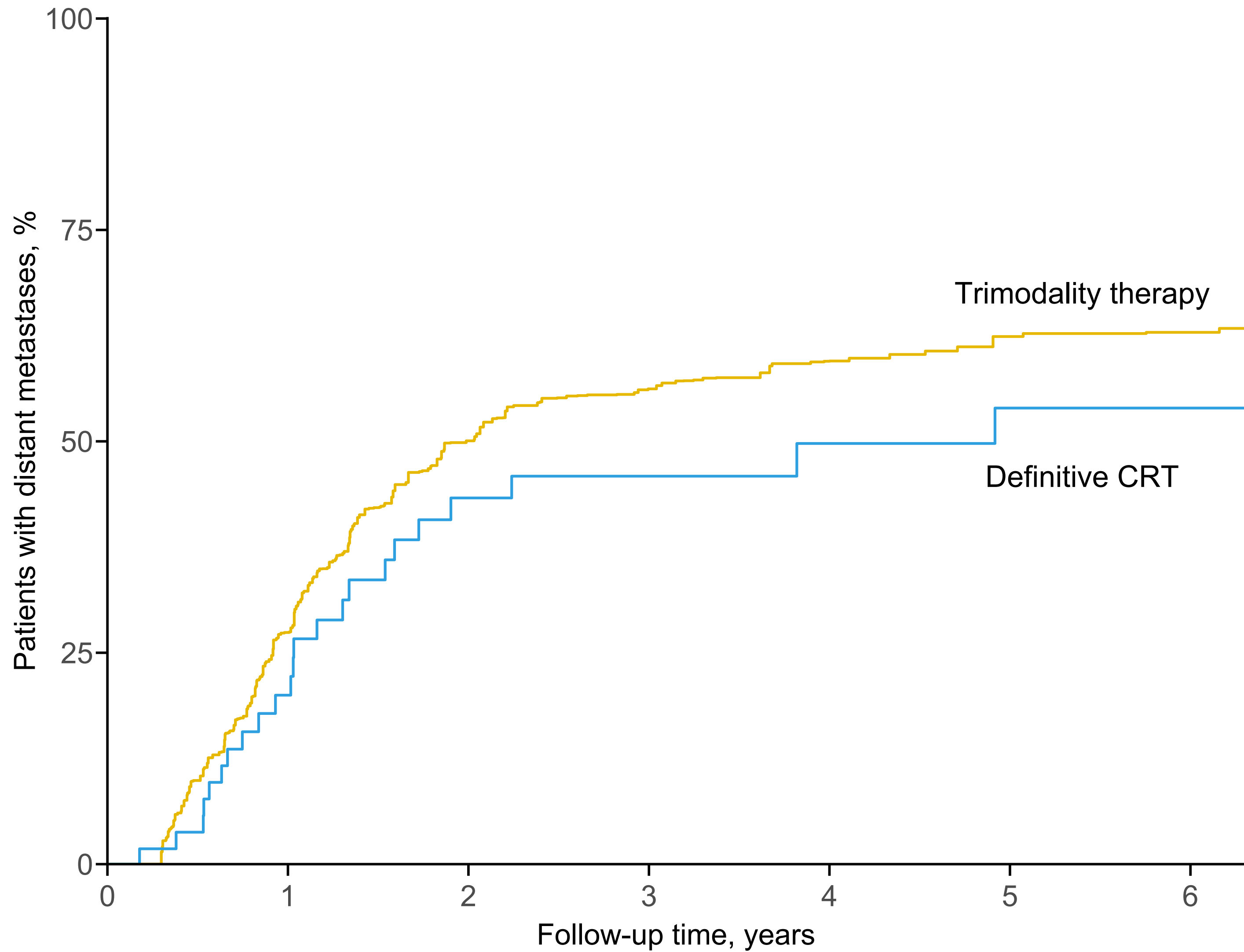

Supplement: Supplementary file 1 [file cancers-16-02850-s001.zip › Figures/DMF_pooled-eps-converted-to.pdf]

**B**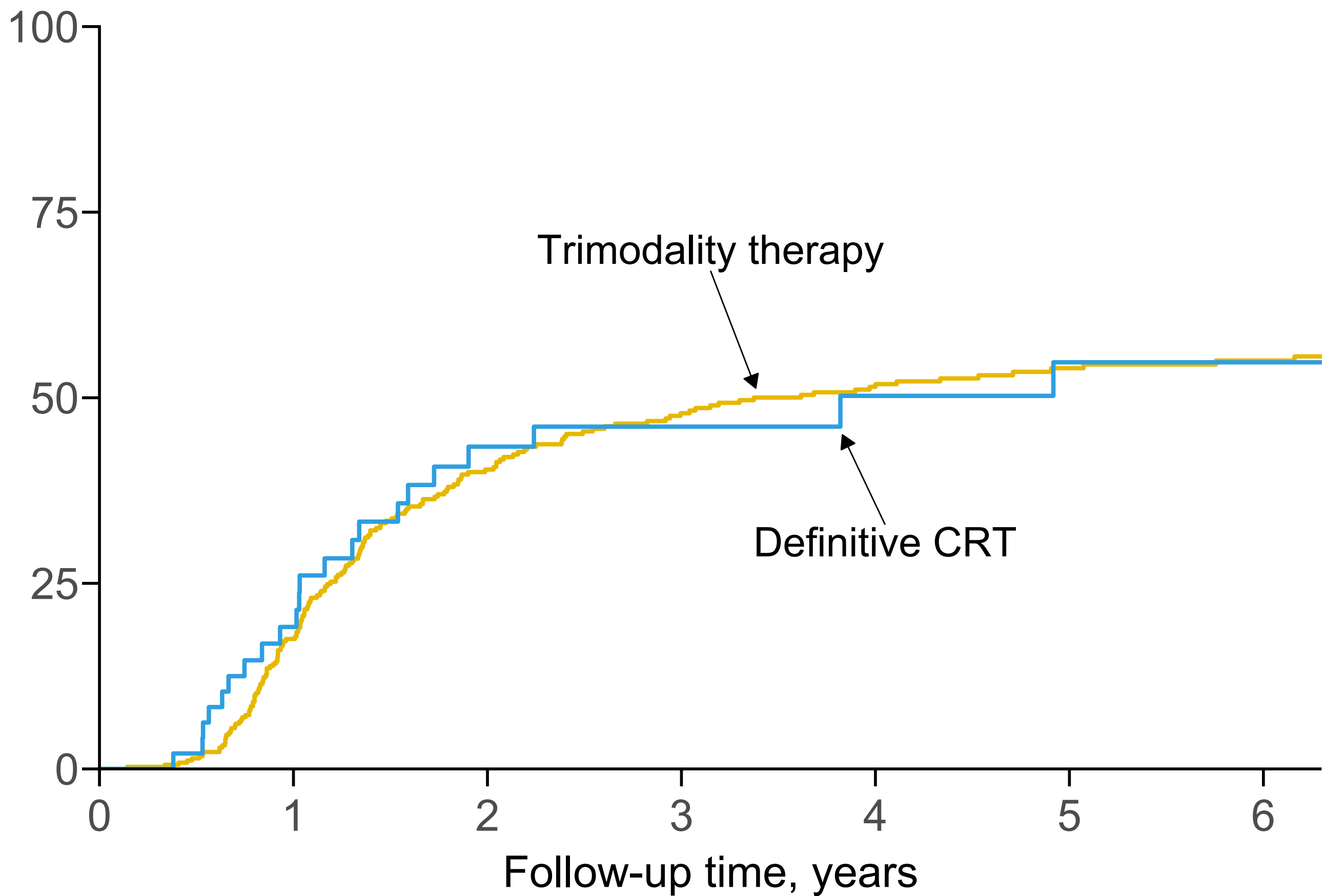

357

269

177

148

132

94

83

49

35

21

13

12

10

8

406

304

198

161

144

104

91

Supplement: Supplementary file 1 [file cancers-16-02850-s001.zip › Figures/DMF_PP_abb-eps-converted-to.pdf]

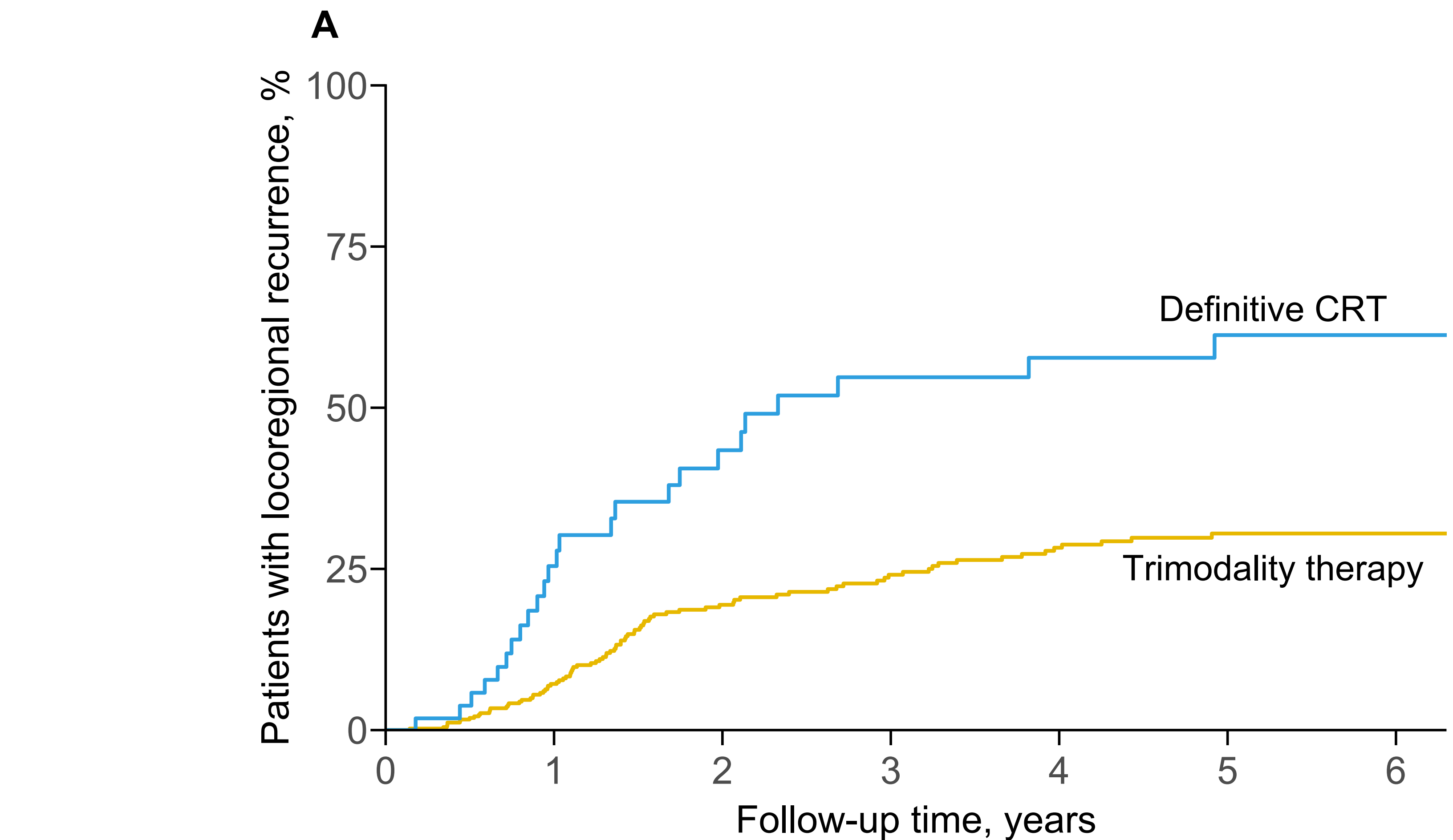

| No. at risk         |     |     |     |     |     |     |    |
|---------------------|-----|-----|-----|-----|-----|-----|----|
| Trimodality therapy | 435 | 326 | 208 | 168 | 148 | 103 | 86 |
| Definitive CRT      | 56  | 31  | 20  | 15  | 13  | 11  | 8  |
| Total               | 491 | 357 | 228 | 181 | 161 | 114 | 94 |

Supplement: Supplementary file 1 [file cancers-16-02850-s001.zip › Figures/LRF-eps-converted-to.pdf]

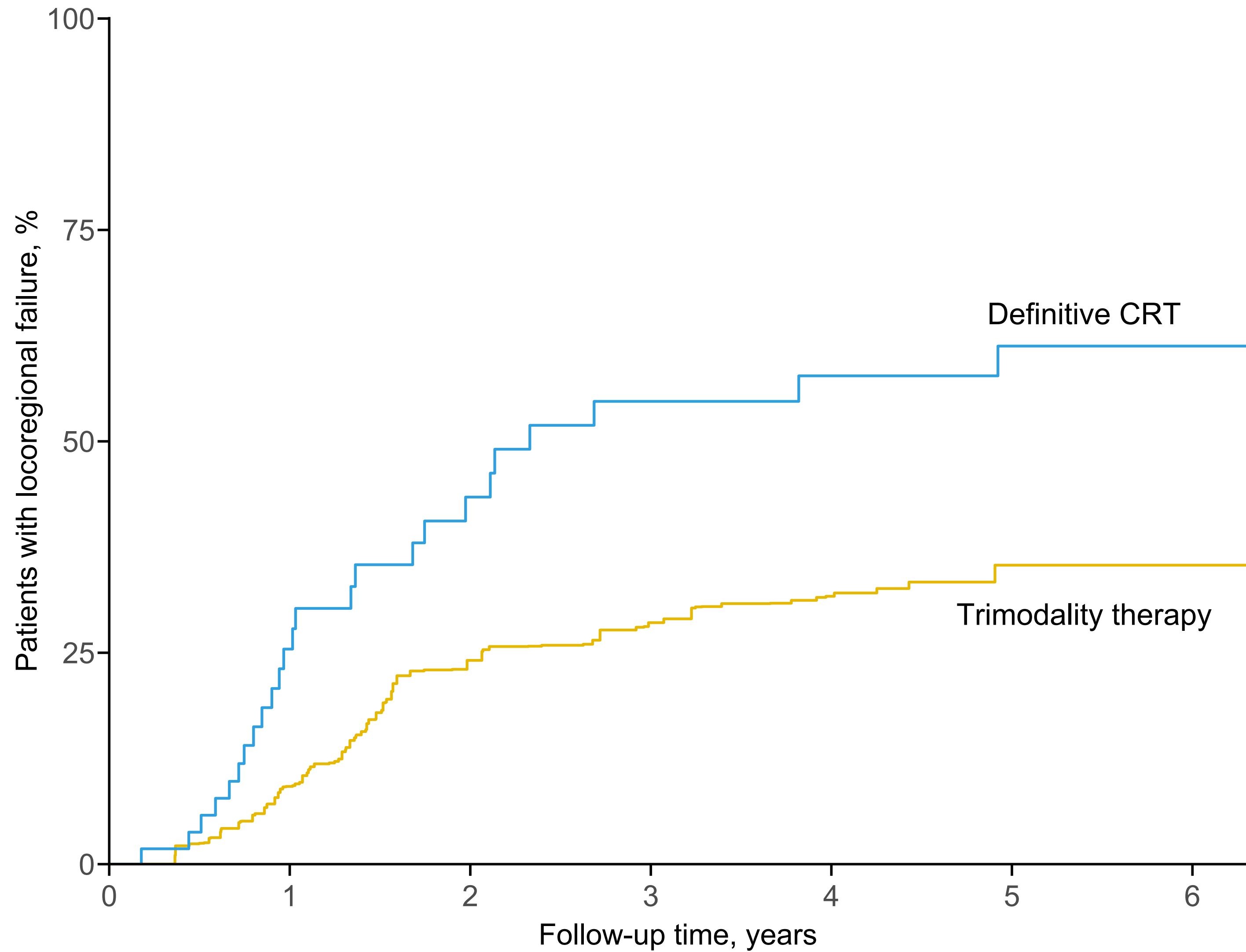

Supplement: Supplementary file 1 [file cancers-16-02850-s001.zip › Figures/LRF_pooled-eps-converted-to.pdf]

**B**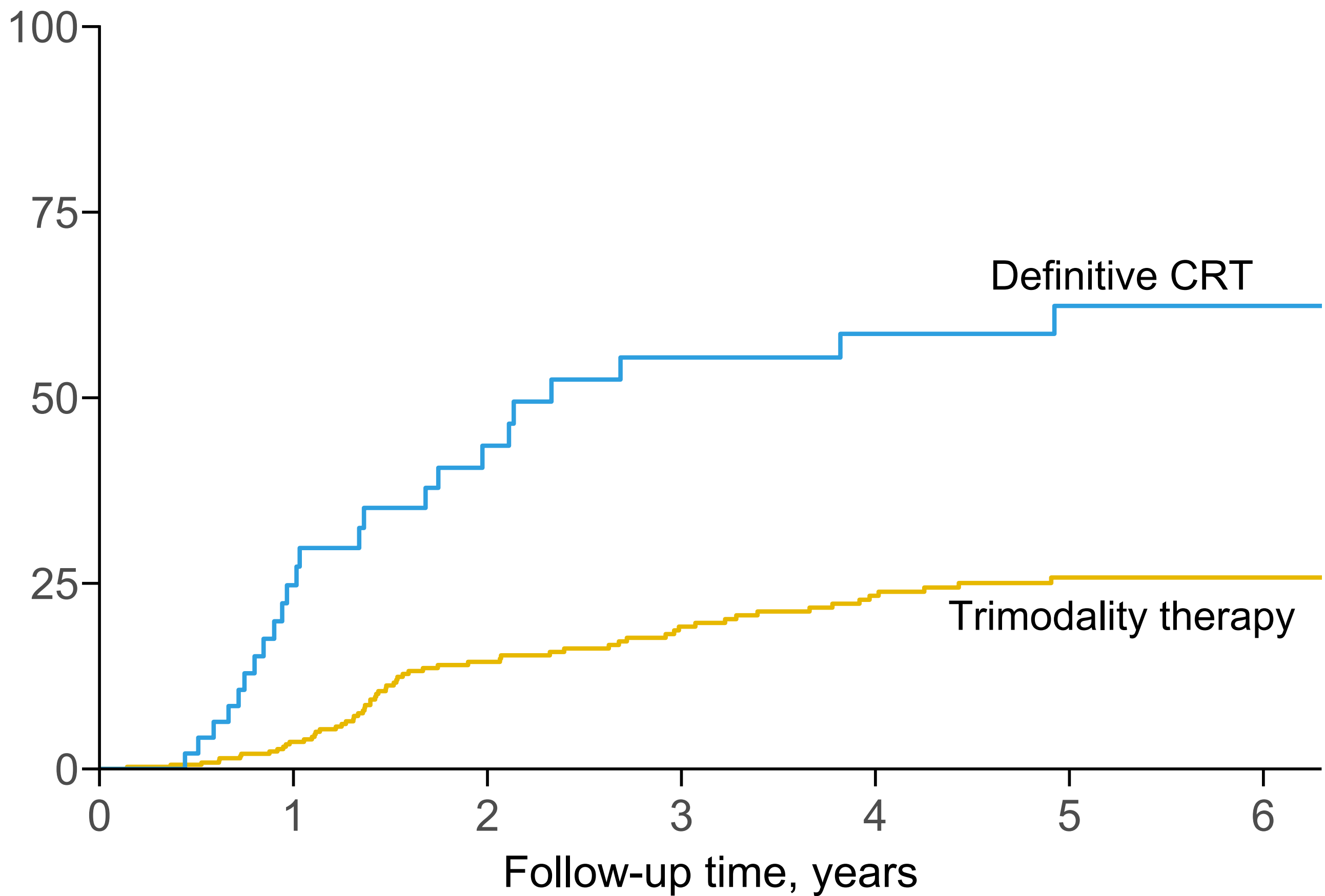

357

291

195

161

142

98

83

49

30

19

14

12

10

8

406

321

214

175

154

108

91

Supplement: Supplementary file 1 [file cancers-16-02850-s001.zip › Figures/LRF_PP_abb-eps-converted-to.pdf]

# Covariate Balance

Range across imputations

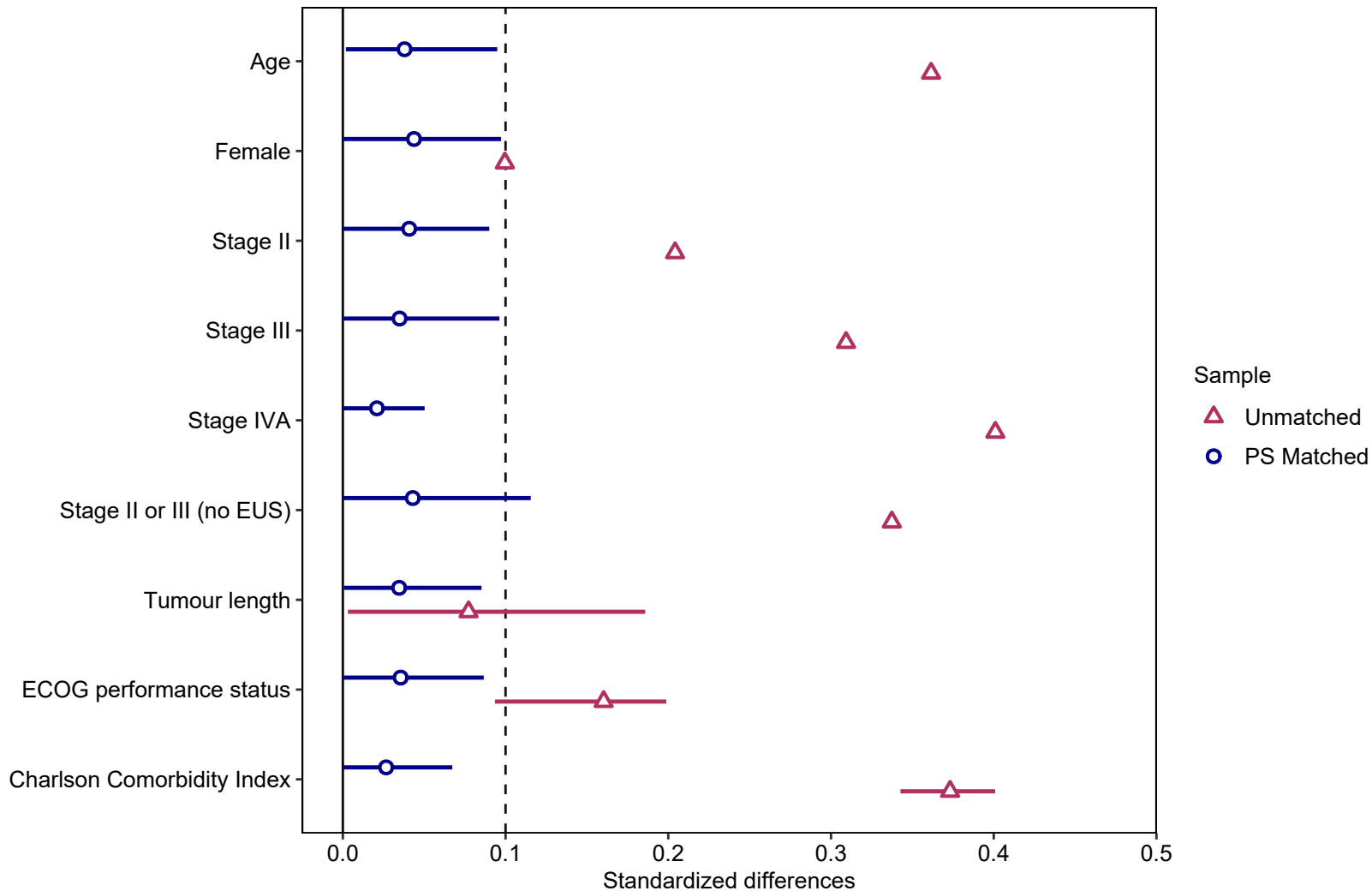

Supplement: Supplementary file 1 [file cancers-16-02850-s001.zip › Figures/match_absolute_love_plot-eps-converted-to.pdf]

# Covariate Balance

Range across imputations

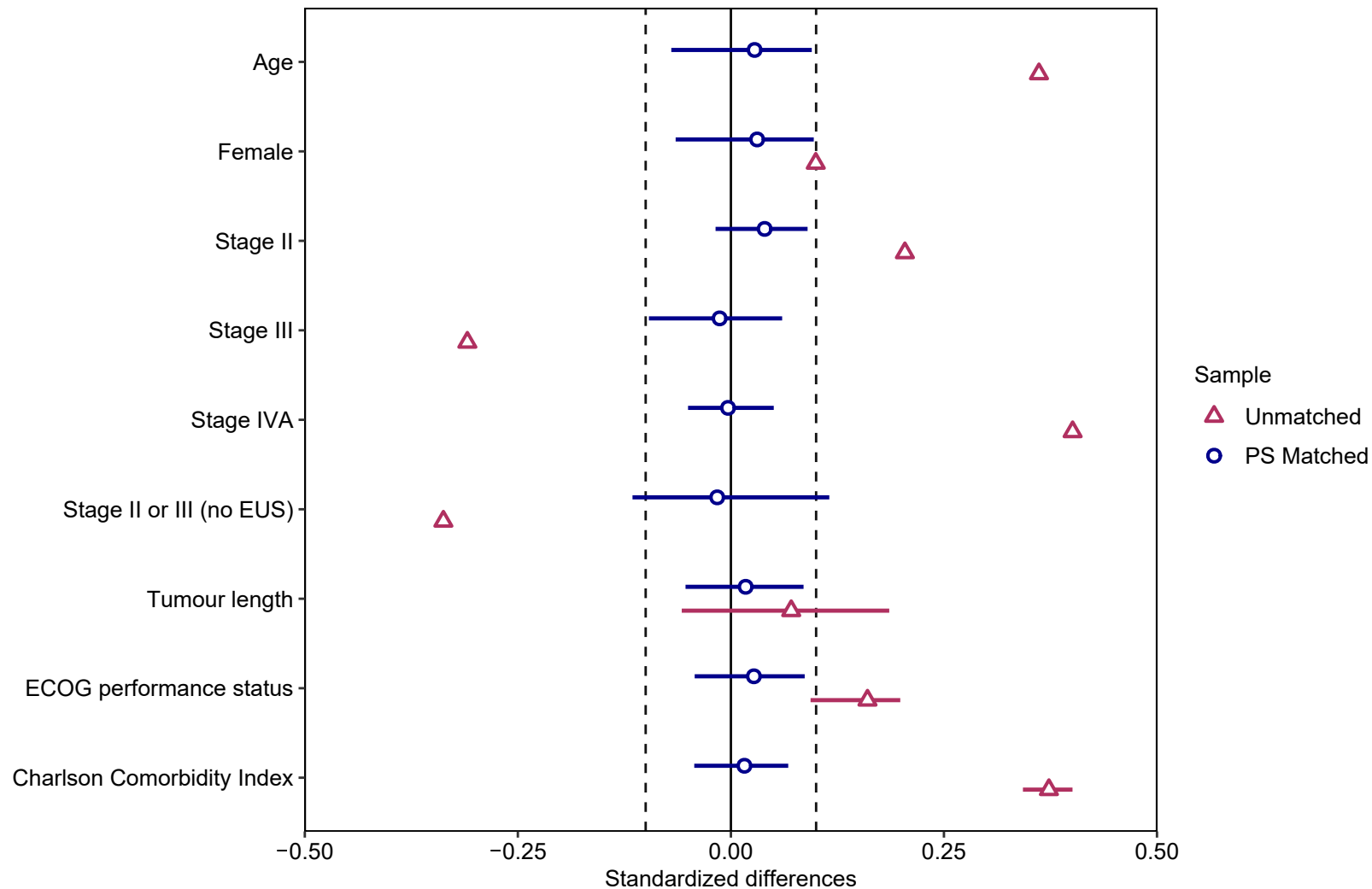

Supplement: Supplementary file 1 [file cancers-16-02850-s001.zip › Figures/match_love_plot-eps-converted-to.pdf]

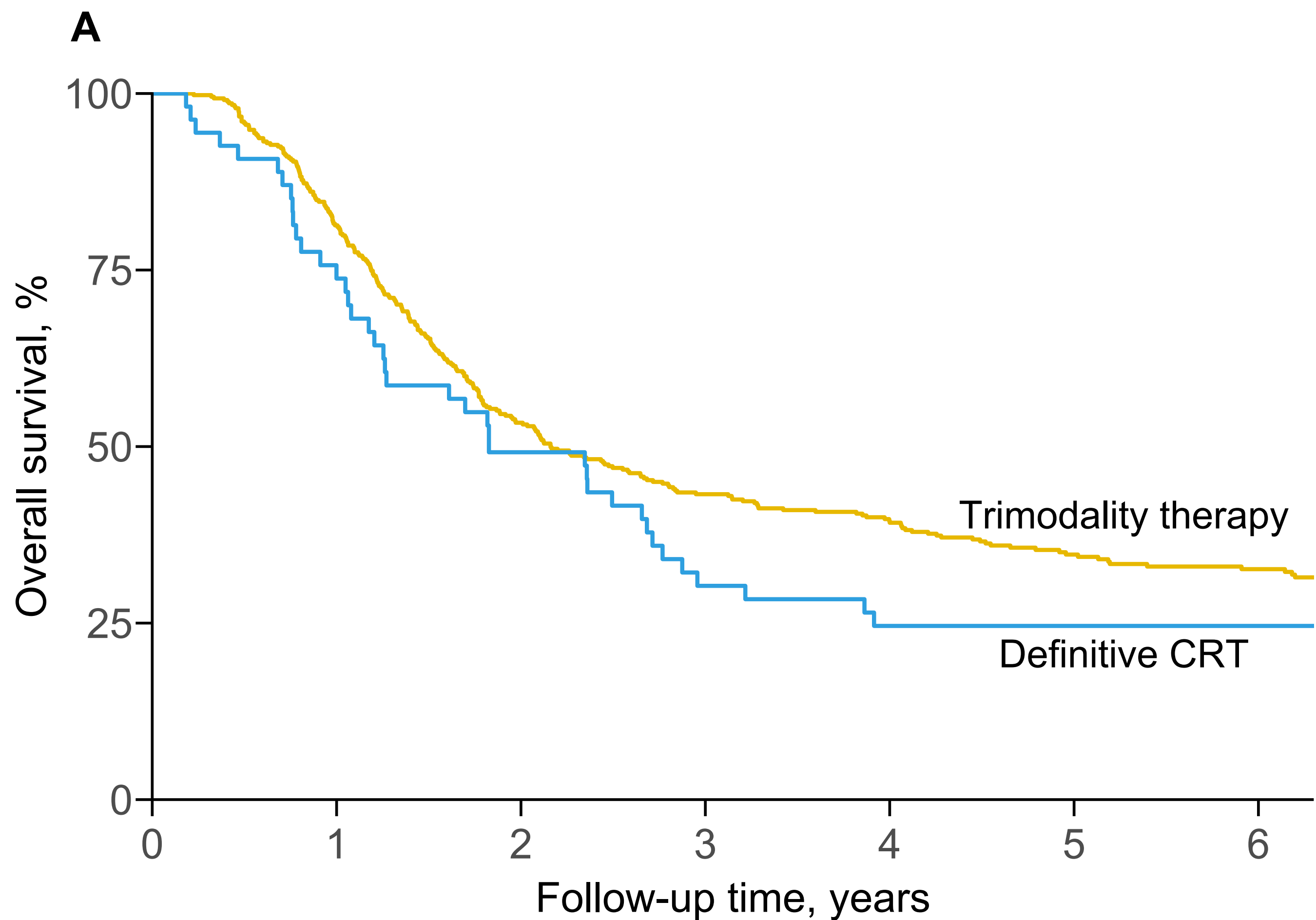

**No. at risk**

|                     |     |     |     |     |     |     |    |
|---------------------|-----|-----|-----|-----|-----|-----|----|
| Trimodality therapy | 435 | 341 | 217 | 173 | 153 | 106 | 87 |
| Definitive CRT      | 56  | 39  | 26  | 16  | 13  | 12  | 9  |
| Total               | 491 | 380 | 243 | 189 | 166 | 128 | 96 |

Supplement: Supplementary file 1 [file cancers-16-02850-s001.zip › Figures/OS-eps-converted-to.pdf]

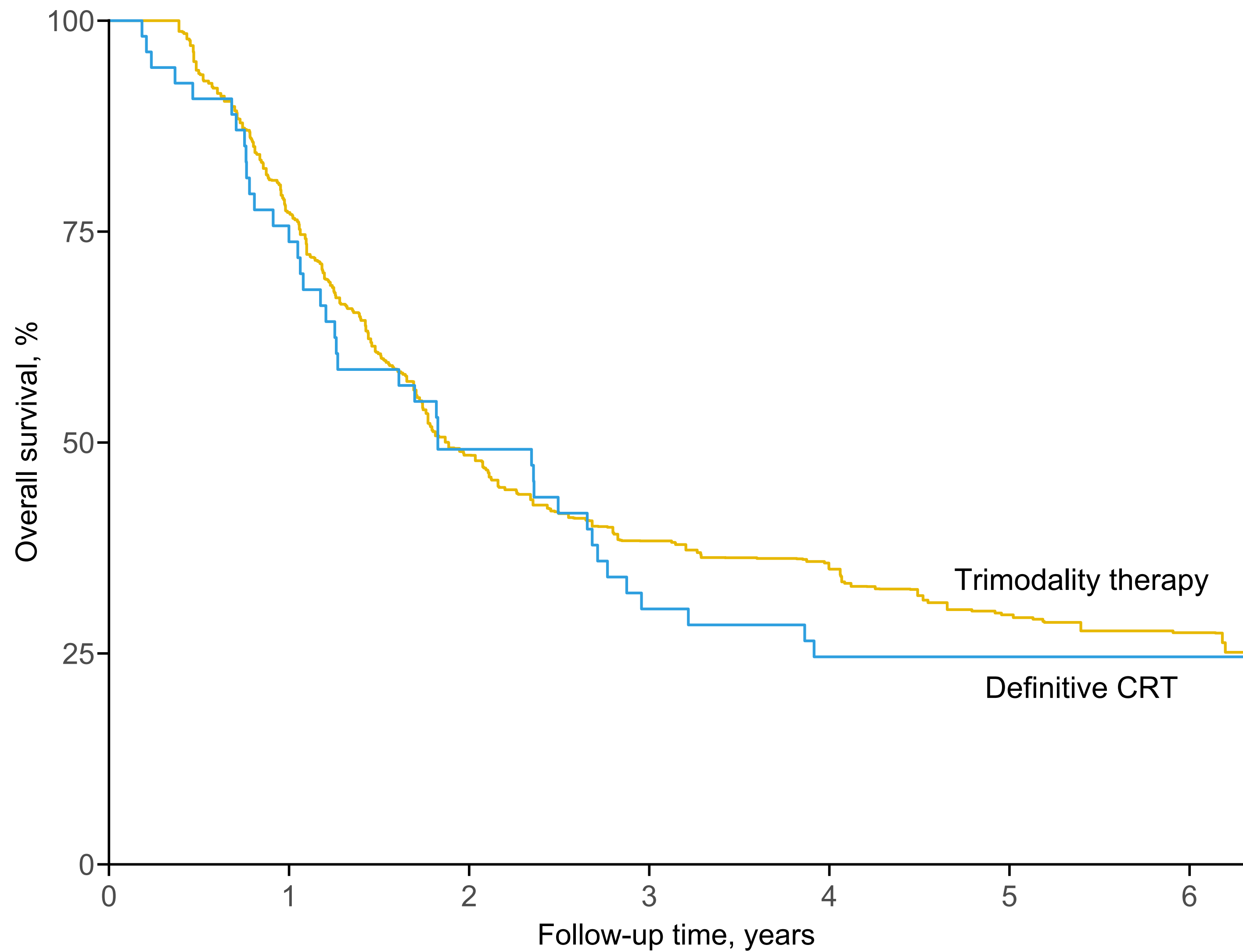

Supplement: Supplementary file 1 [file cancers-16-02850-s001.zip › Figures/OS_pooled-eps-converted-to.pdf]

**B**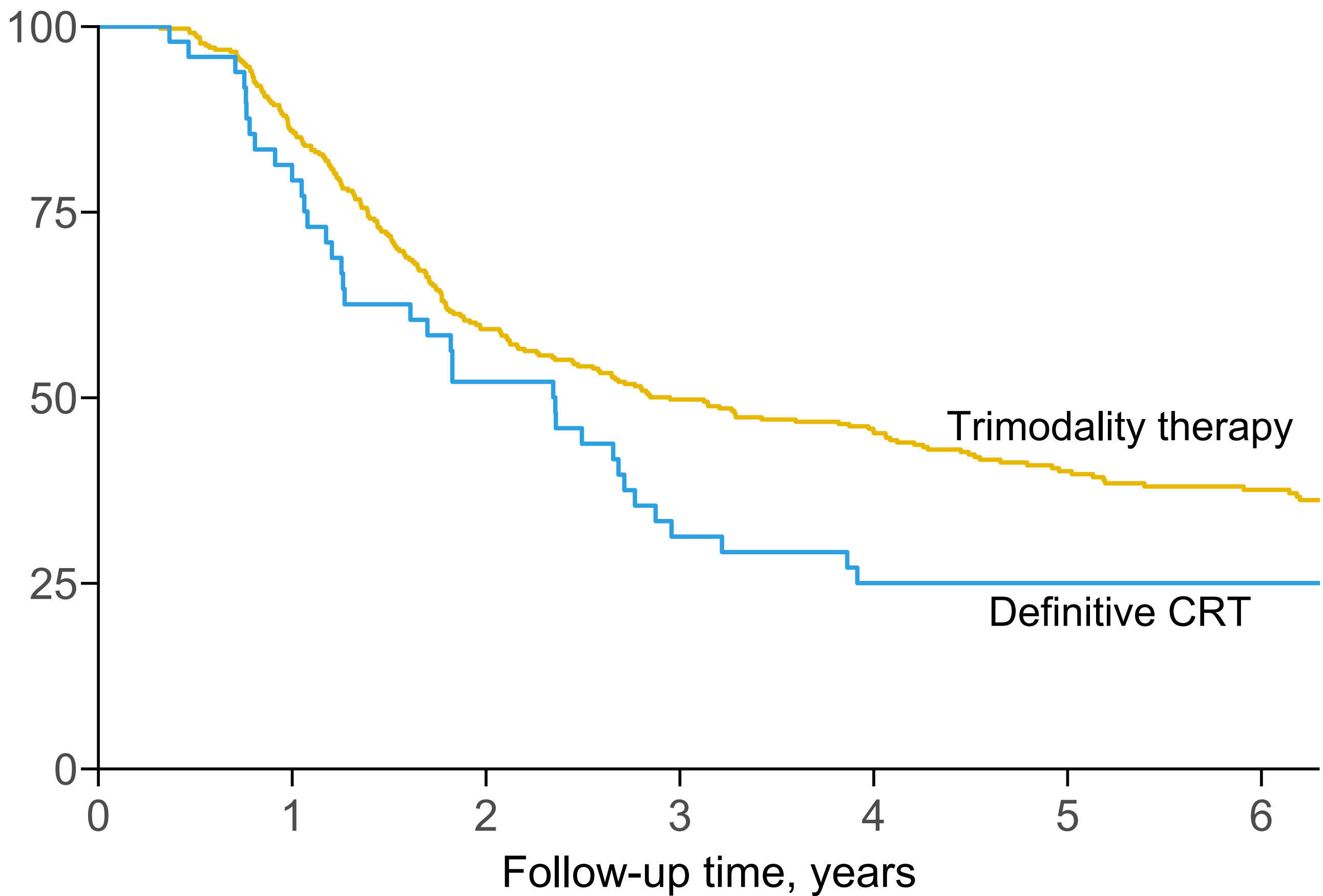

357

298

201

166

147

101

84

49

38

25

15

12

11

9

406

336

226

181

159

122

93

Supplement: Supplementary file 1 [file cancers-16-02850-s001.zip › Figures/OS_PP_abb-eps-converted-to.pdf]

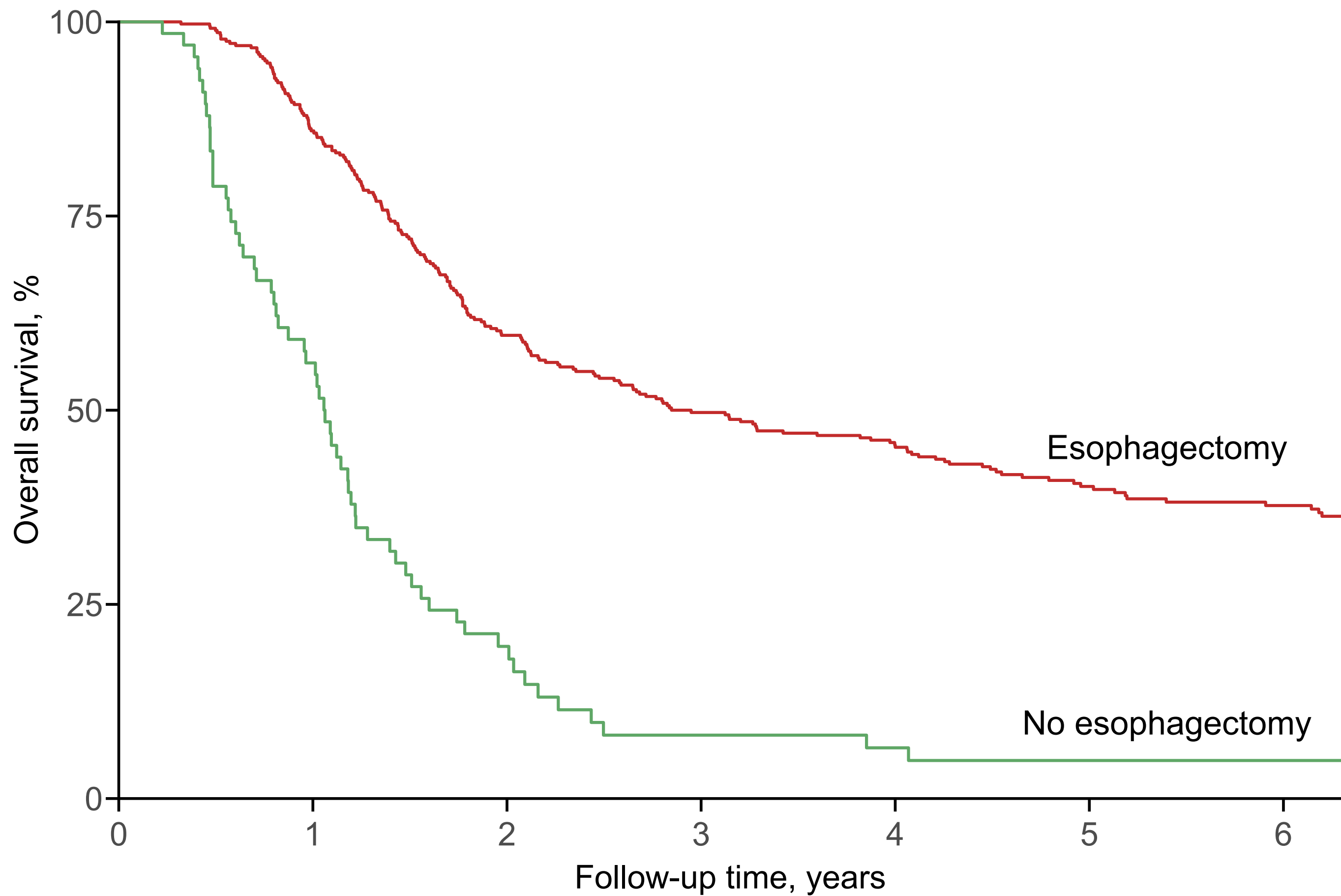

| No. at risk      |     |     |     |     |     |     |    |
|------------------|-----|-----|-----|-----|-----|-----|----|
| Esophagectomy    | 368 | 304 | 205 | 168 | 149 | 103 | 85 |
| No esophagectomy | 67  | 37  | 12  | 5   | 4   | 3   | 2  |
| Total            | 435 | 341 | 217 | 173 | 153 | 106 | 87 |

Supplement: Supplementary file 1 [file cancers-16-02850-s001.zip › Figures/OS_trimodal_intent_surgery-eps-converted-to.pdf]
